# Phase Stability and Structural Reorganization of Silica in Cherts Under Thermal and Mechanochemical Stress

**DOI:** 10.3390/ma18133077

**Published:** 2025-06-28

**Authors:** María de Uribe-Zorita, Pedro Álvarez-Lloret, Beatriz Ramajo, Javier F. Reynes, Celia Marcos

**Affiliations:** 1 Department of Geology, Faculty of Geology, University of Oviedo, C. Jesús Arias de Velasco s/n, 33005 Oviedo, Spain; pedroalvarez@uniovi.es; 2 Scientific-Technical Services, University of Oviedo, 33005 Oviedo, Spain; ramajobeatriz@uniovi.es; 3 Department of Organic and Inorganic Chemistry, Faculty of Chemistry, University of Oviedo, 33006 Oviedo, Spain; fernandezreyjavier@uniovi.es

**Keywords:** chert, silica polymorphs, thermal treatment, mechanochemical treatment

## Abstract

This work investigated the structural response and phase transformation dynamics of silica-bearing cherts subjected to high-temperature processing (up to 1400 °C) and prolonged mechanochemical activation. Through a combination of X-ray diffraction (XRD) with Rietveld refinement, differential scanning calorimetry (DSC), thermogravimetric analysis (TGA), and transmission electron microscopy (HRTEM), we trace the crystallographic pathways of quartz, moganite, tridymite, and cristobalite under controlled thermal and mechanical stress regimes. The experimental results demonstrated that phase behavior is highly dependent on intrinsic properties such as initial phase composition, impurity presence, and crystallinity. Heating at 1400 °C induced irreversible conversion of quartz, moganite, and tridymite into cristobalite. Samples enriched in cristobalite and tridymite exhibited notable increases in crystallinity, whereas quartz-dominant samples showed either stability or a decline in structural order. Rietveld analyses underscored the critical influence of microstrain and crystallite size on thermal resilience and phase persistence. Thermal profiles revealed by DSC and TGA expose overlapping processes including polymorphic transitions, minor phase dehydration, and redox-driven changes, likely associated with trace components. Mechanochemical processing resulted in partial amorphization and the emergence of phases such as opal and feldspar minerals (microcline, albite, anorthite), interpreted as the product of lattice collapse and subsequent reprecipitation. Heat treatment of chert leads to a progressive rearrangement and recrystallization of its silica phases: quartz collapses around 1000 °C before recovering, tridymite emerges as an intermediate phase, and cristobalite shows the greatest crystallite size growth and least deformation at 1400 °C. These phase changes serve as markers of high-temperature exposure, guiding the identification of heat-altered lithic artefacts, reconstructing geological and diagenetic histories, and allowing engineers to adjust the thermal expansion of ceramic materials. Mechanochemical results provide new insights into the physicochemical evolution of metastable silica systems and offer valuable implications for the design and thermal conditioning of silica-based functional materials used in high-temperature ceramics, glasses, and refractory applications. From a geoarchaeological standpoint, the mechanochemically treated material could simulate natural weathering of prehistoric chert tools, providing insights into diagenetic pathways and lithic degradation processes.

## 1. Introduction

**Figure 1:**
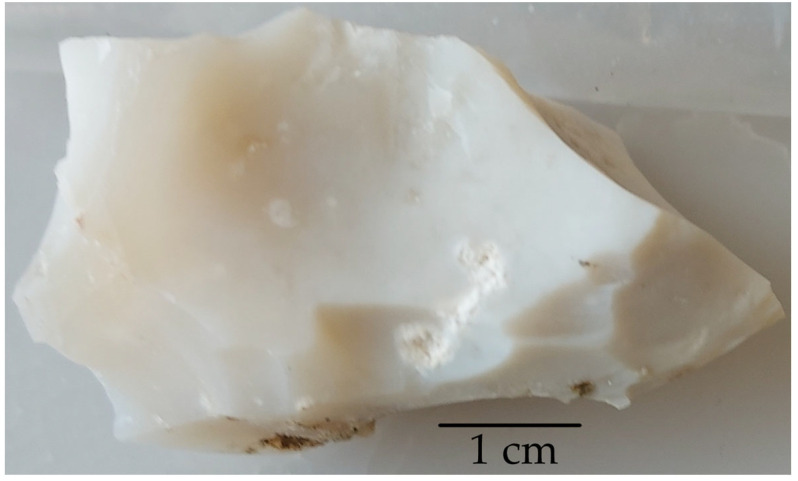
Textural appearance of a chert (Madrid, Spain) in hand specimen.

Chert is a fine-grained siliceous sedimentary rock (Figure 1) composed primarily (>90%) of microcrystalline or cryptocrystalline forms of SiO_2_, such as quartz and moganite [1,2]. Although typically uniform in texture and mineralogy, chert exhibits a variety of colors and often contains microfossils, including diatoms, radiolarians, and sponge spicules [3,4]. Its geological occurrence spans from the Precambrian to the Quaternary, forming in bedded or nodular bodies intercalated with carbonate rocks [5,6,7].

The origin of silica in chert has been linked to both abiotic precipitation and biogenic contributions [3,4]. During the Archean and Paleoproterozoic (4000–1600 Ma), silica precipitated directly from seawater [3], whereas in later periods such as the Neoproterozoic (1600–541 Ma) and Cenozoic (66 Ma to present), biological sources (e.g., siliceous sponges and diatoms) played a dominant role [3,5,6,7,8,9]. Consequently, the mineralogical maturity and diagenetic pathways of chert vary depending on its formation environment and age.

At atmospheric pressure, silica polymorphs include quartz, tridymite, and cristobalite, each displaying reversible α–β transitions associated with minor structural adjustments; the transformation from quartz α to quartz β occurs at 573 °C, the transformation from tridymite α to tridymite β occurs at ≈265 °C, and the transformation from cristobalite α to cristobalite β occurs between 200 and 270 °C [10,11]. In contrast, reconstructive transitions between these polymorphs involve major framework reorganization due to the breaking and reforming of Si–O–Si linkages, occurring at elevated temperatures and requiring substantial thermal energy [12,13,14]. Classical phase diagrams indicate the quartz–tridymite–cristobalite transition sequence beginning near 870 °C [10], although natural systems often deviate due to kinetic barriers, stacking faults, or the presence of catalytic impurities such as Al^3+^ [15,16].

In chert, high-temperature transformation behaviors differ significantly depending on mineral assemblage and microstructure. Quartz can undergo structural collapse at elevated temperatures, with partial recrystallization occurring upon further heating [13,14,17]. Tridymite and cristobalite often coexist, sometimes within a single crystal, and show variations in peak profiles and reflection intensities in X-ray diffraction (XRD) due to stacking disorder [18,19,20]. Moganite, a lesser-known silica polymorph commonly coexisting with quartz in chert, undergoes a reversible phase transition from monoclinic to orthorhombic symmetry between 296 and 316 °C [21,22] and converts to cristobalite at 900–1000 °C [23,24]. Moganite does not appear to undergo pressure-induced transformations [25] and is associated with evaporitic and hydrothermal systems [22,26]. Its role as an intermediate phase in the diagenetic sequence opal-A → opal-CT → moganite → quartz has been documented in both geothermal and fossilization contexts [27,28,29].

Mechanochemical processing—via milling or grinding—has emerged as a powerful alternative to thermal activation in modifying silica phases. It induces structural disorder, polymorphic transitions, and even amorphization through stress-driven reorganization, offering a low-energy and sustainable route for material transformation [30,31,32,33]. In natural cherts, this may simulate diagenetic conditions or prefigure anthropogenic modifications such as those seen in ceramic processing.

In addition to its geological and archaeological significance, heat-treated chert has potential as a functional raw material in high-performance ceramics and glass-ceramics. The formation of high-temperature silica polymorphs such as β-cristobalite and tridymite, as observed in thermally transformed cherts, plays a pivotal role in tuning the thermal expansion properties of ceramic bodies, improving compatibility with glazes and reducing the risk of cracking. Dapiaggi et al. [34] showed that such transformations can be modulated by alkali content, grain size, and thermal conditions—parameters that may be naturally embedded in chert. Therefore, studying the structural evolution of chert under elevated temperatures not only advances our mineralogical understanding but also opens avenues for its valorization in industrial applications where controlled thermal behavior is critical.

Chert is a compositionally complex and often heterogeneous material, in contrast to the pure or synthetically doped silica phases typically used in phase transition studies. This inherent variability is precisely one of the motivations behind our choice to work with natural cherts: to explore real-world systems with geological and archaeological relevance. The chert samples selected for this study were chosen from among those available to us according to the following criteria: (1) the availability of material suitable for the full set of experimental analyses that we wanted to carry out, (2) diversity in chert type, and (3) variety of geological and geographical origins. Differences in minor and trace element contents (e.g., Al_2_O_3_, MgO) among the samples, as discussed in the literature, can act as structural stabilizers or transformation catalysts for tridymite and cristobalite [15,16].

The present study aims to characterize the crystallographic and thermal behavior of silica polymorphs in chert under thermal stress, at 1000 °C and 1400 °C, and mechanochemical activation. Using X-ray diffraction (XRD) with Rietveld refinement, thermogravimetric analysis (TGA), differential scanning calorimetry (DSC), and transmission electron microscopy (HRTEM), we assess transformation sequences involving quartz, tridymite, cristobalite, and moganite, including their structural stabilization and potential amorphization. Our findings contribute to a better understanding of silica phase stability in geologic and engineered systems and inform the design of silica-based materials for high-temperature and stress applications.

## 2. Materials and Methods

### 2.1. Materials and Experimental Treatments

Several chert samples MN34 and MN35 (Montana, USA), LB (Libourne, France), ULL (Ulldemolins in Tarragona, Spain), MB and MBE (Getafe in Madrid, Spain), and CAL (Vicálvaro in Madrid, Spain) were treated at 1000 °C and at 1400 °C. Samples treated at 1000 °C were coded as MN34-1000, MN35-1000, LB-1000, ULL-1000, MB-1000, MBE-1000, and CAL-1000; samples treated at 1400 °C were coded as MN34-1400, MN35-1400, LB-1400, ULL-1400, MB-1400, MBE-1400 and CAL-1400. Two chert, CG (Cabo de Gata, Almería, Spain), a quartzitic chert, and MBE, a cristobalite–tridymite chert, were selected for mechanochemical experiments. Each material was milled for 4, 8, 12, 16, 20, and 24 (h), and the products were labelled CG-4, CG-8, CG-12, CG-16, CG-20, CG-24 and MBE-4, MBE-8, MBE-12, MBE-16, MBE-20, MBE-24.

The thermal treatment was made using the raw chert samples (MN34, MN35, LB, ULL, MB, MBE, CAL) in a Carbolite RFH1600 electrical furnace (Hope Valley, UK). The heating process was programmed to increase from room temperature to 1000 °C at a rate of 5 °C/min, under restricted oxygen conditions, meaning they were placed in open aluminum crucibles without any sealing or covering. Upon reaching 1000 °C, the samples were maintained at this temperature for 48 h. Finally, the furnace was allowed to cool down slowly to room temperature at a cooling rate similar to the heating rate. The heating process was programmed under the same conditions for 1400 °C.

The mechanochemical treatments were made with Retsch MM500 Vario (Sheffield, UK) equipment, with up to six grinds at a time. The frequency was set at 30 Hz from 4 h to 24 h, 4 h at a time, using a 440C stainless steel Retsch screw closure jar of 10 mL with thin PTFE seal and one 10 mm-diameter 440C stainless steel milling ball. The mill and balls used in the mechanochemical treatment are composed of stainless steel 440B, which is a high-carbon, martensitic stainless steel with the dual advantages of high hardness and good corrosion resistance. Reasons why stainless steel 440B is suitable for chert treatment include the following: High carbon content (around 0.75–0.95% C) and high chromium content (around 17%) make this steel capable of achieving high hardness and excellent wear resistance. It has a martensitic structure, which responds well to surface modification processes. Stainless steel 440B is particularly suitable for chert treatment due to its metallurgical properties. Its high carbon content (approximately 0.75–0.95%) and elevated chromium level (around 17%) allow it to reach high hardness and excellent wear resistance, both critical for processing hard siliceous materials like chert. Furthermore, its martensitic microstructure responds well to surface modification techniques, enhancing its performance in demanding mechanical and thermal conditions.

### 2.2. Characterization

The characterization of the samples was performed using a combination of X-ray fluorescence and X-ray diffraction (XRD), thermogravimetric analysis (TGA), and differential scanning calorimetry (DSC). XRD analysis was employed to identify and quantify the crystalline phases, while TGA and DSC were used to monitor thermal responses in the analyzed samples. The chemical composition of the samples was determined using X-ray fluorescence with a Shimadzu EDX-720 energy dispersive X-ray fluorescence spectrometer (XRF-EDX) (Kyoto, Japan). This model is equipped with five types of filters for reducing and eliminating background, characteristic lines, and other types of scattered radiation; collimator, 10 mm; voltage 50 kV and current 40 μA; measurement time was 100 s. The optimum calibration curve for the sample is selected automatically from pre-registered calibration curves.

XRD patterns were obtained using a PANalytical X’Pert Pro MPD diffractometer (Almelo, The Netherlands), operating at 45 kV and 40 mA with Cu Kα radiation (λ = 1.5418 Å). Measurements were conducted over a 2θ range of 5–70°, with a step size of 0.007° and a counting time of 1 s per step. Phase identification, quantification, and determination of crystallographic parameters were conducted using X’Pert HighScore Plus v2.2d (2.24, 2008), applying the Rietveld refinement method. Initial phase identification was conducted using the PDF-2 and ICSD databases. Crystallinity was assessed by calculating the ratio of the integrated intensities of crystalline diffraction peaks to the total measured intensity using X’Pert HighScore Plus v2.2d (2.24, 2008), according to Equation (1):

(1) $$Crystallinity\left(\%\right)=100\sum I_{net.}/\left(\sum I_{tot.}-\sum I_{const.bgr.}\right)$$

where

Σ*I_net_* is the sum of the net peak intensities, which are the integrated intensities of the crystalline diffraction peaks, i.e., the portion of the signal associated with crystalline phases in the sample. Each intensity is measured after subtracting the background under the peak. This reflects how much of the XRD signal comes from ordered, crystalline domains.

Σ*I_tot_* is the total intensity sum. This is the sum of all measured intensities across the entire XRD pattern, including both peaks and background. It represents the total diffracted signal, encompassing both crystalline and amorphous contributions.

Σ*I_const.bgr_*_._ is the constant background intensity. This term refers to the constant component of the background signal, often due to scattering from amorphous phases, the sample holder, or instrument noise. It is subtracted from *I_tot_* to eliminate non-structural contributions. This correction ensures that only structurally meaningful signal (crystalline + variable amorphous) is considered. After determining the background intensity and separating crystalline peaks from the amorphous hump, the software automatically calculates the crystallinity percentage. Although no internal standard was used to quantify the amorphous fraction explicitly, the crystallinity values obtained act as an effective proxy for estimating the relative degree of amorphization. Lower crystallinity percentages in thermally or mechanically treated samples indicate a higher proportion of disordered or amorphous silica. This indirect method, while not yielding absolute amorphous content, enables consistent comparison across samples with varying composition and diagenetic maturity.

High-resolution transmission electron microscopy (HRTEM) with JEOL-JEM-2100F (Akishima, Tokyo, Japan) operated 200 kV, with a vacuum of 1.0 × 10−5 Pa, was used to study the crystallographic structure of the samples, which were analyzed using transmission mode and the selected area electron diffraction (SAED) mode of that electron microscope. For the analysis, a few drops of the suspension of each powdered sample (in ethanol) were dispersed on a carbon-coated copper grid. The samples analyzed by transmission electron microscopy (TEM and HRTEM) were LB-1000 and MN34-1000.

Thermal behavior of the samples was further investigated using a TA Instruments SDT Q600 simultaneous TGA–DSC analyzer (New Castle, DE, USA). Each sample was heated from room temperature to 1375 °C at a constant rate of 5 °C/min under an oxygen flow of 100 mL/min. Prior to each experiment, the instrument was calibrated following the manufacturer’s specifications to ensure data accuracy and reproducibility. The resulting thermal signals were processed using TA Instruments Universal Analysis 2000 software.

To interpret thermal effects and structural changes, statistical analyses were conducted using IBM SPSS Statistics version 24 (IBM Corp., Armonk, NY, USA). These included one-way analysis of variance (ANOVA) to evaluate differences in crystallinity under distinct thermal treatments, and principal component analysis (PCA) to explore the influence of compositional factors on crystallinity development. PCA also allowed us to assess clustering trends among the samples, potentially linked to their diagenetic histories.

## 3. Results

The composition of the samples investigated is presented in Table 1. Silica (SiO_2_) content in the analyzed cherts ranges from 87.87% to 99.97%, distinguishing high-purity quartzitic samples (e.g., MN35, LB) from those with notable impurities (e.g., MB, MBE). Al_2_O_3_ levels are highest in ULL (9.43%), indicating clay-rich input, while MgO is significant in MBE (8.01%), linked to the presence of sepiolite. K_2_O and Fe_2_O_3_, when present, suggest minor contributions from phyllosilicates and iron oxides. Loss on ignition (L.O.I.) values, ranging from 1.89% to 4.78%, reflects variable amounts of volatile components, organics, carbonates, sulfates. These chemical profiles support a distinction between recrystallized, silica-rich cherts and impurity-bearing types influenced by detrital or diagenetic phases [35].

### 3.1. Heat-Treated Cherts at 1000 °C and 1400 °C

**Figure 2:**
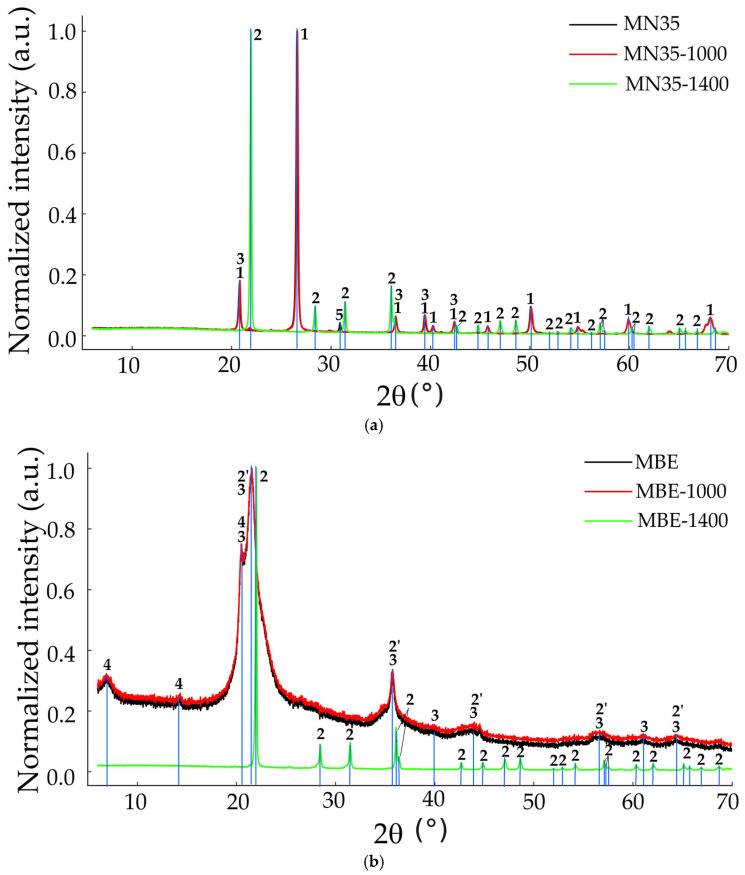

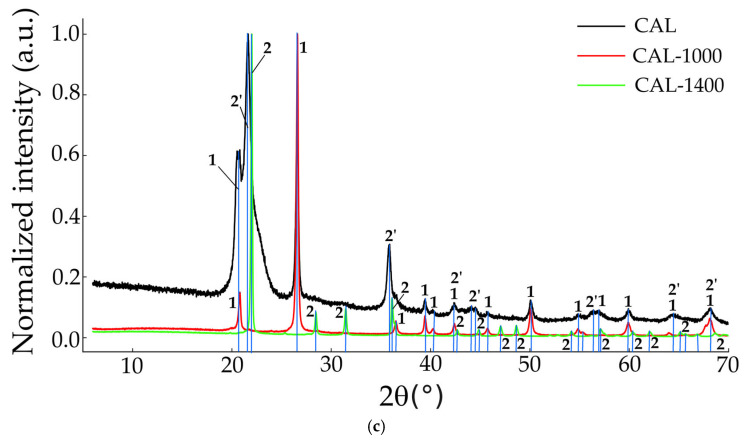
X-ray diffraction patterns of the untreated cherts and cherts heated at 1000 °C and 1400 °C of the samples MN35 (**a**), MBE (**b**), and CAL (**c**), as examples, showing the principal reflections of the identified phases. In the sample NM35, 1 = quartz (100), (101), (110), (102), (111), (201), (112), (103), (211), (203) plus moganite overlapping with quartz; 2 = cristobalite tetragonal (101), (111), (102), (112), (211), (202), (113), (212), (203), (311), (302), (312), (214), (105); 3 = tridymite overlapping with quartz reflections; 5 = dolomite (104). In the sample MBE, 3 = trydimite (112), ($\bar{4}$04), (020), (606), (822), (824), (137); 2 = cristobalite tetragonal with the same reflections as NM35; 2′ = cristobalite cubic (111), (220); 4 = sepiolite ($\bar{1}21$), (214). In the sample CAL, 1 = quartz with the same reflections as NM35; moganite overlapping with quartz; 2= cristobalite tetragonal with the same reflections as NM35; 2′ = cristobalite cubic (111), (220), (331) plus reflections—(311), (422), (511)—overlapping with those of quartz.

In Table 2 the phases identified and their corresponding ICDD card in the investigated samples are presented. Figure 2 displays only the representative X-ray diffraction patterns of samples MN35, MBE, and CAL, because MN34 and ULL replicate the MN35 pattern, MB mirrors MBE, and LB behaves like CAL; the samples MN34, ULL, MB, and LB are shown in Figure S1 (Supplementary Materials). In cherts heated to 1400 °C, only reflections corresponding to cristobalite were observed. This indicates that quartz, moganite, and tridymite were totally transformed into cristobalite at 1400 °C. X-ray diffraction analysis revealed that all raw samples contain quartz as the primary crystalline phase, often accompanied by moganite, except in the MN35 and MBE samples. These quartz–moganite assemblages are characteristic of well-recrystallized cherts. In contrast, cristobalite and tridymite were dominant in MBE and MB, especially after thermal treatment, indicating transformation of less-ordered silica phases.

A comprehensive structural analysis was performed using Rietveld refinement of X-ray diffraction data on chert samples: untreated, treated at 1000 °C, and treated at 1400 °C. The focus was exclusively on the four main silica phases: quartz, moganite, tridymite, and cristobalite (Table S1: Rietveld data), although minor amounts of non-silica phases were detected through Rietveld refinement, namely sepiolite (0.1%) in sample MBE and dolomite in MN35 (1.6%) and MN34 (0.2%), which may contribute locally to the observed thermal behavior. The quality of the Rietveld refinements was evaluated using the goodness-of-fit (GOF) parameter, which ranged from 5 (e.g., MN35-1400, MBE-1400) to 14 (CAL-1000) across the analyzed samples, with intermediate values such as 8 (LB-1000, MB-1000) and 12 (CAL, MB, MN35). Structural data from the following cif files were used for the refinement: amcsd-0000789 (low-quartz), amcsd-0006370 (high-quartz), amcsd-0002737 (moganite), amcsd-0020937 (hexagonal tridymite), amcsd-0020733 (orthorhombic tridymite), amcsd-0017665 (cubic cristobalite), amcsd-0001629 (tetragonal cristobalite). The thermal evolution of the chert samples reveals a clear, data-driven sequence of silica-phase transformations. Normalized averages show that quartz dominates the untreated material (43.4%) but increases to 44.8% at 1000 °C and disappears entirely at 1400 °C. Moganite, initially present at 2.7%, rises transiently to 10.4% at 1000 °C—reflecting sample-specific retention—but is no longer detectable after exposure to 1400 °C, confirming its well-known metastability. Tridymite nucleates at 1000 °C with a mean content of 13.6% (unit-cell parameters a ≈ 17.8 Å, b ≈ 5.08 Å, c ≈ 24.8 Å; V ≈ 2154 Å^3^; crystallite size ≈ 116 Å) and vanishes at 1400 °C, having fully converted to cristobalite. Cristobalite displays the most pronounced growth: it accounts for 28.0% in the untreated cherts, increases to 31.2% at 1000 °C, and becomes the sole silica phase (100%) at 1400 °C. Concomitantly its lattice contracts from a ≈ 5.01 Å, c ≈ 7.11 Å (V ≈ 187 Å^3^) to a ≈ 4.971 Å, c ≈ 6.925 Å (V ≈ 171 Å^3^) while its crystallite size quadruples (≈ 227 Å → ≈822 Å), indicating advanced recrystallization and microstrain relaxation.

**Figure 3:**
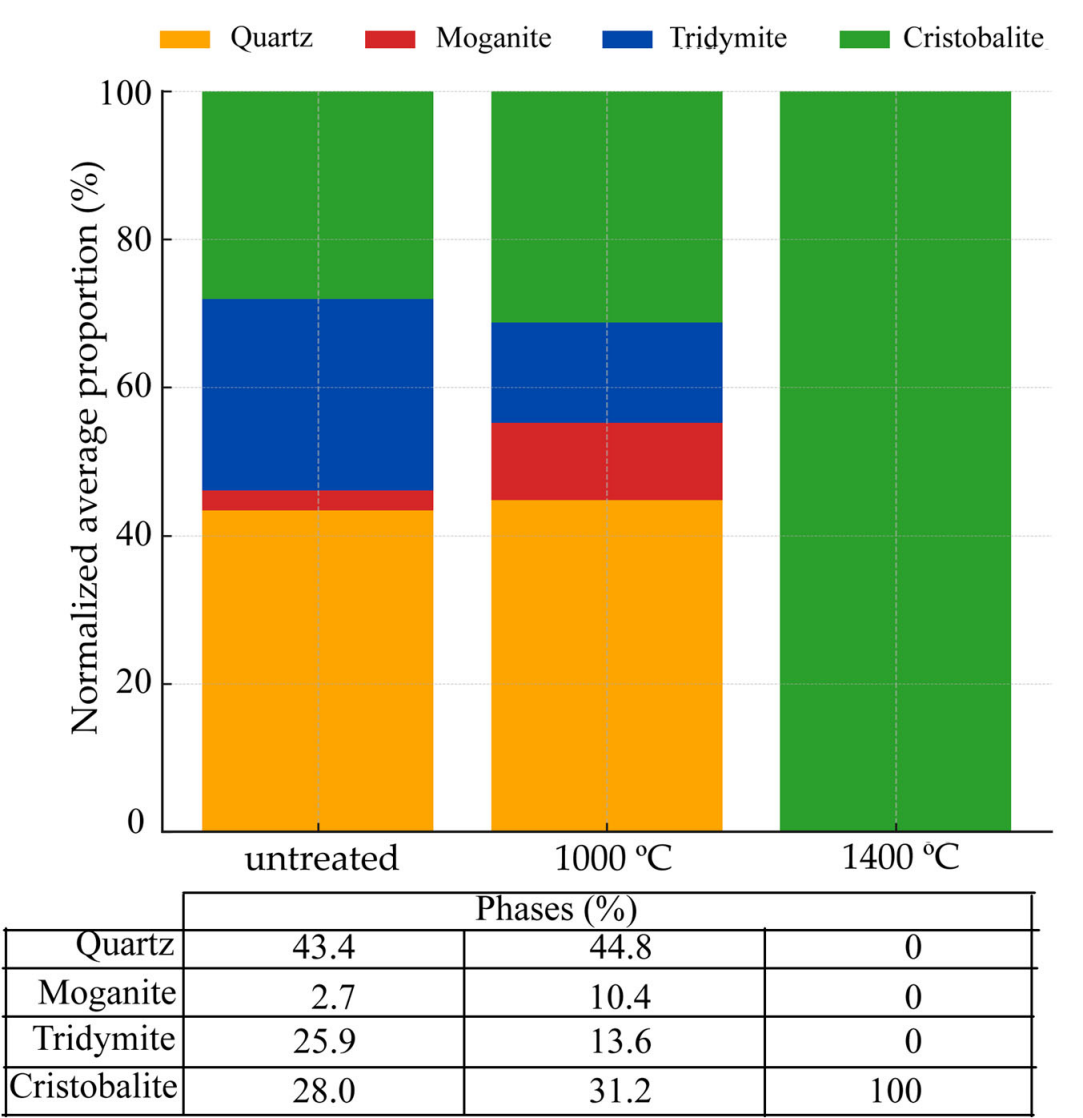
Evolution of silica phases in chert samples subjected to progressive thermal treatment. Values represent the normalized average proportion of each phase—quartz, moganite, tridymite, and cristobalite—calculated independently for untreated samples and those heated to 1000 °C and 1400 °C. A table below the graph summarizes the normalized average percentage content of the four major silica phases identified in chert samples under each thermal treatment condition. Percentages represent phase normalized averages of silica phases across samples (excluding non-silica components).

The phase transformations in the silica components of chert samples subjected to thermal treatment at 1000 °C and 1400 °C can be seen in Figure 3. The comparative table below the graph summarizes these quantitative changes, facilitating the visual interpretation of thermally induced mineralogical transitions. Quartz remained the dominant phase in both the untreated samples and those heated to 1000 °C. Notably, moganite increased in average proportion from 2.7% to 10.4% at 1000 °C, likely due to improved detectability following structural reordering. Tridymite and cristobalite were already present in the untreated samples, with tridymite decreasing from 25.9% to 13.6% and cristobalite increasing slightly from 28.0% to 31.2% at 1000 °C. These trends reflect the progressive stabilization of cristobalite under thermal treatment, while tridymite becomes less abundant.

Crystallinity values for the analyzed cherts are summarized in Table 3. The analysis revealed significant behaviors in the crystallinity of the samples subjected to thermal treatment. The variations in crystallinity changes may be attributed to the initial compositions of the samples, the nature of the minerals present, or their specific responses to heat treatment. Samples with lower initial crystallinity were more vulnerable to significant positive changes, while those with higher initial crystallinity tended to remain stable or slightly decrease. All samples showed increased crystallinity after 1400 °C treatment. Samples with lower initial crystallinity showed the highest percentage increases. There is a positive correlation between treatment temperature and final crystallinity.

The combination of chemical, mineralogical, and thermal crystallinity data revealed significant structural differences among the analyzed cherts. High-silica samples (e.g., LB, CAL, MN34, MN35) exhibited stable quartz–moganite assemblages with high initial crystallinity and minimal structural changes upon heating. Conversely, samples with higher Al_2_O_3_ or MgO contents (e.g., MB, MBE, ULL) showed low initial crystallinity.

Statistical analysis revealed significant differences in crystallinity across thermal treatments. Pairwise comparisons showed *p*-values below 0.05 for both untreated vs. 1400 °C and 1000 °C vs. 1400 °C, confirming that the heat exposure induced meaningful structural changes in the cherts. High-silica samples and quartzitic samples, such as LB (65.9%), MN35 (70.2%), and MN34 (69.1%) showed high initial crystallinity and minor changes after heating. In contrast, cristobalitic/tridymitic samples such as MB (31.2%), MBE (26.3%), and 16,681 (51.6%) had low initial values but showed sharp increases at 1400 °C—up to 75.8%, 70.7%, and 72.5%, respectively. These shifts correlate with the formation of cristobalite and tridymite in impurity-rich samples. ANOVA confirmed significant differences between mineralogical groups at all temperatures, supporting the distinction between quartzitic and cristobalitic/tridymitic cherts.

**Figure 4:**
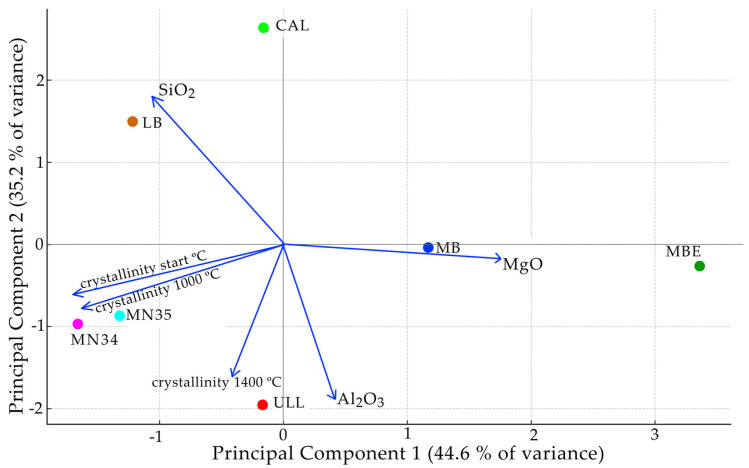
Biplot of the PCA graphic showing the direction and magnitude (blue arrows) of variable contributions (SiO_2_, Al_2_O_3_, MgO) on the positioning of the samples in the PCA space. The longer the arrow, the stronger the influence on the data distribution. Arrows pointing in similar directions indicate variables are positively correlated. Arrows in opposite directions indicate negative correlation.

A principal component analysis (PCA) (Figure 4) was applied to identify compositional controls (SiO_2_, Al_2_O_3_, MgO) on crystallinity values (untreated, 1000 °C, and 1400 °C) and to distinguish sample groupings linked to diagenetic processes. The PCA results support the above-mentioned observations, distinguishing silica-rich from impurity-driven samples. SiO_2_ content correlated negatively with structural transformation, while Al_2_O_3_ and MgO were positively associated with thermal crystallization. The first two principal components explained 82.7% of the total variance. Samples with high silica content clustered together, whereas those enriched in Al_2_O_3_ or MgO appeared separated due to their distinct thermal behavior.

To further corroborate the silica phase transformations identified by XRD and Rietveld refinement, selected chert samples subjected to heating at 1000 °C were analyzed using high-resolution transmission electron microscopy (HRTEM) and selected area electron diffraction (SAED). This complementary technique allowed for nanoscale identification of individual crystalline domains and provided direct lattice-resolved evidence of the mineral phases present at this intermediate thermal treatment stage, where multiple silica polymorphs coexist.

**Figure 5:**
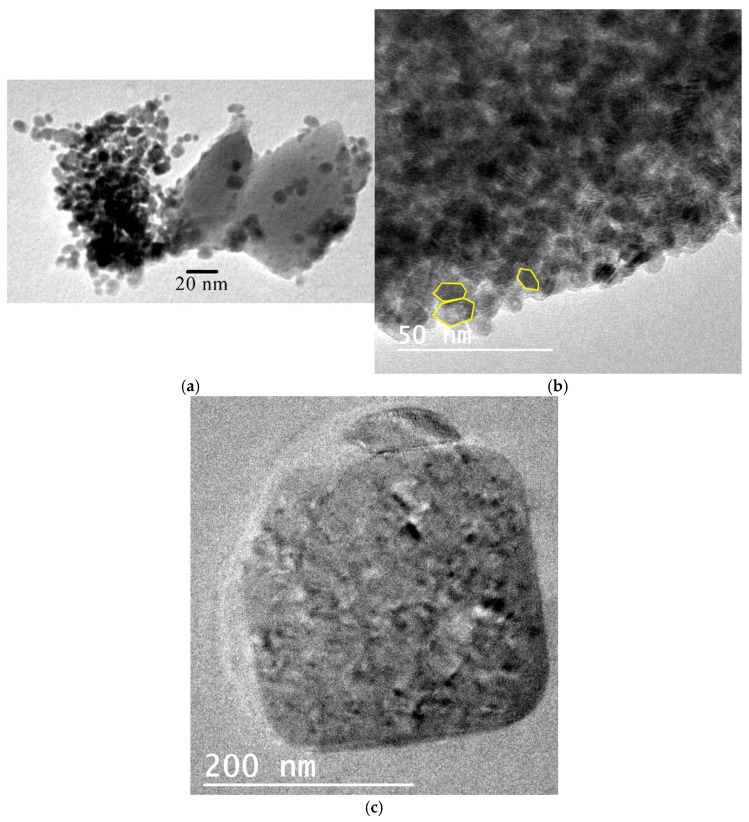
Images HRTEM showing habits of nano crystals: (**a**) rounded appearance in LB-1000 sample, (**b**) pseudo-hexagonal habit (attributable to quartz and tridymite) in MN34-1000 sample, and (**c**) almost square-shaped habit (typical of cristobalite) in MN34-1000 sample.

HRTEM imaging of samples LB-1000 and MN34-1000 revealed nanocrystals with characteristic morphologies: rounded forms, pseudo-hexagonal outlines (attributable to quartz and tridymite), and near-square habits (typical of cristobalite) (Figure 5).

**Figure 6:**
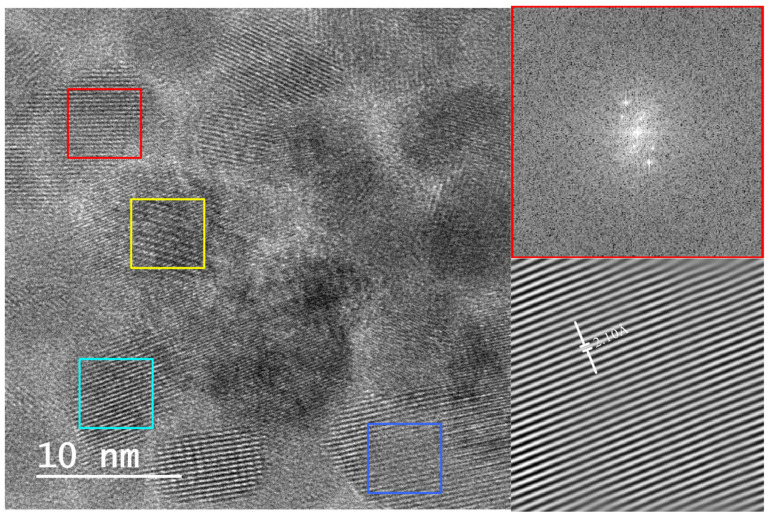

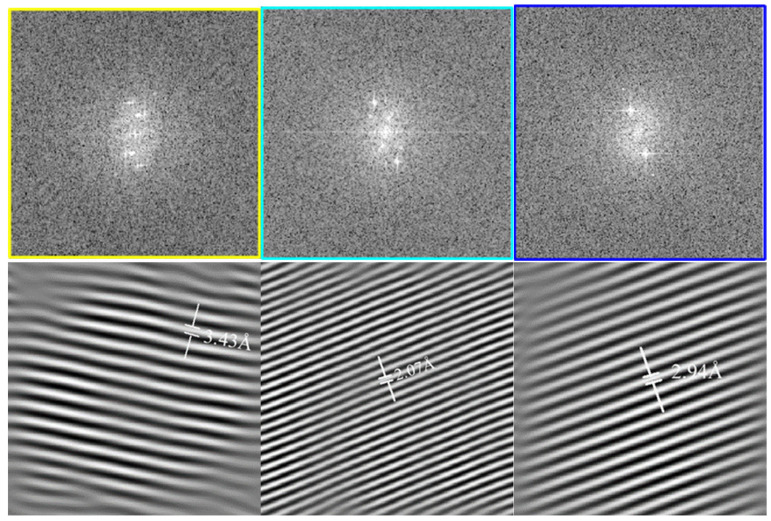
HRTEM image of lattice fringes from LB-1000 sample showing different interplanar distances (colored squares) and their analyzed Fourier transforms that confirmed the presence of tridymite (red, 2.10 Å; turquoise blue, 2.07 Å; dark blue, 2.94 Å) and cristobalite (yellow, 3.43 Å).

**Figure 7:**
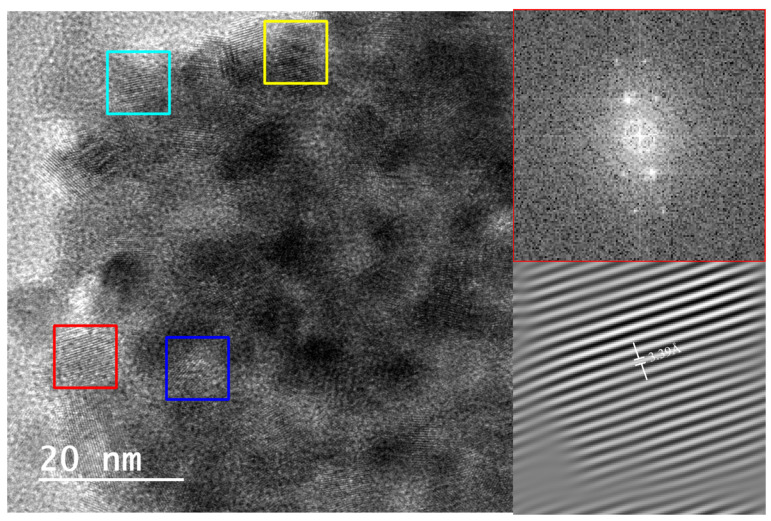

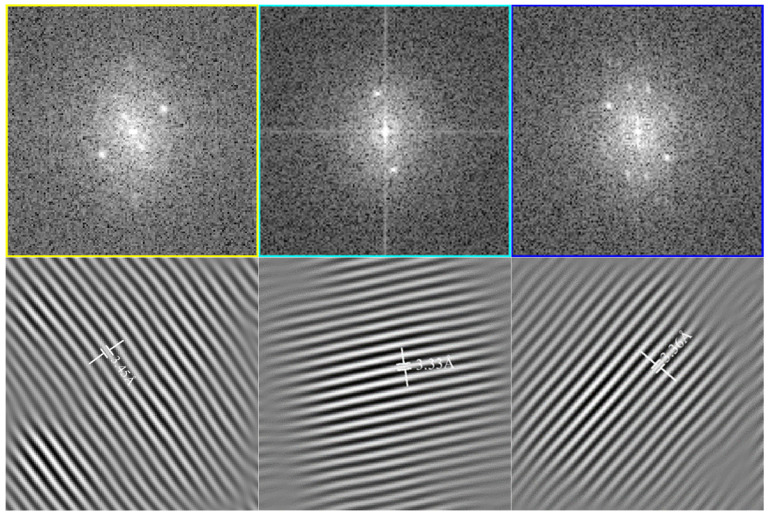
HRTEM image of lattice fringes from MN34-1000 sample showing different interplanar distances (colored squares) and their analyzed Fourier transforms that confirmed the presence of moganite (red, 3.39 Å); yellow, 3.36 Å; dark blue, 3.33 Å) or quartz (dark blue, 3.33 Å) and cristobalite (turquoise blue, 3.45 Å).

**Figure 8:**
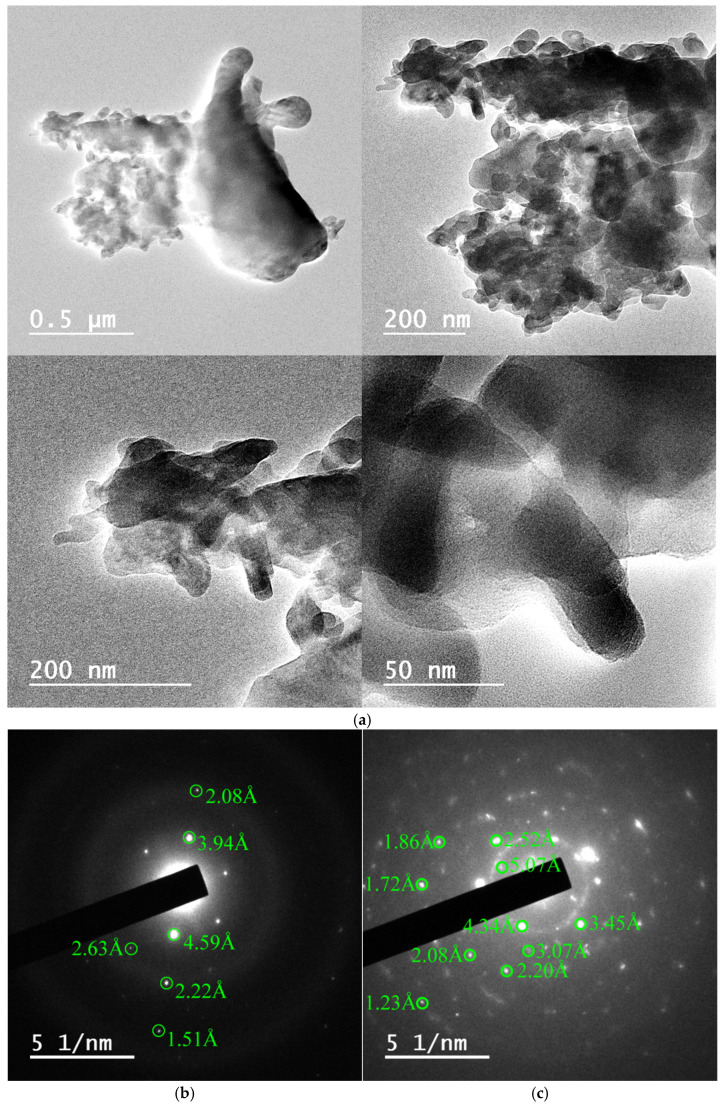
(**a**) HRTEM micrographs of sample LB-1000 showing their layered structure; (**b**,**c**) their corresponding SAED patterns showing a single diffraction pattern corresponding to tridymite (**b**) and polycrystalline-like diffraction pattern with interplanar distances corresponding to tridymite, cristobalite, and moganite (**c**).

**Figure 9:**
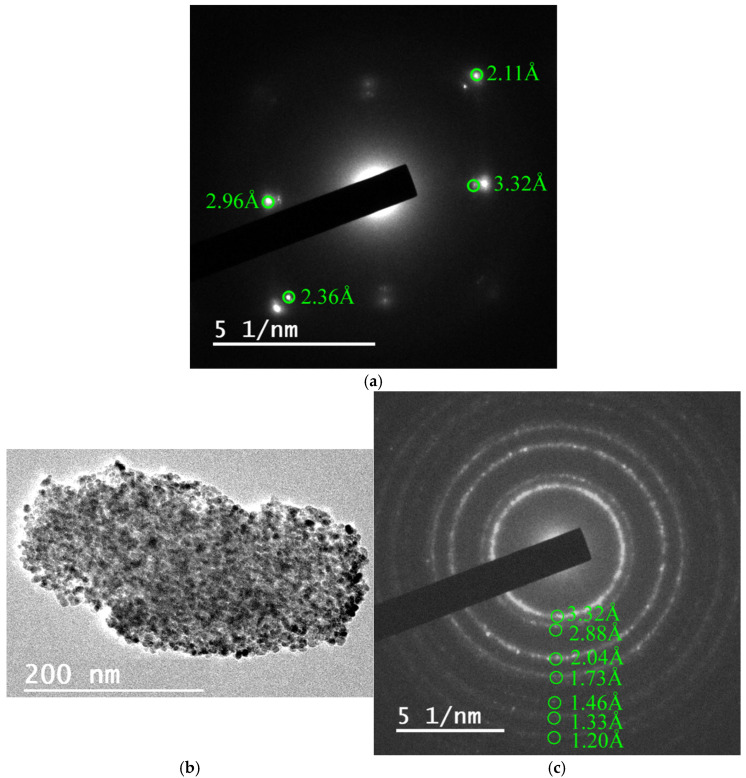
(**a**) SAED pattern of a crystal from sample MN34-1000 (Figure 8c) showing the reflection (101) of quartz and the reflections of tridymite; (**b**) HRTEM micrograph of sample MN34-1000 showing a polycrystalline particle; (**c**) SAED pattern with d*_hkl_* (3.32, 1.73, 1.46, 1.20 Å) attributable to quartz, d*_hkl_* (2.88, 1.46, 1.20 Å) attributable to cristobalite (AMSCD 20170), and d*_hkl_* (2.04, 1.33 Å) attributable to tridymite (AMSCD 13132).

Lattice fringe measurements and corresponding FFT (fast Fourier transform) patterns confirmed the presence of tridymite (2.10–2.94 Å), cristobalite (3.43–3.45 Å), quartz (3.32–3.33 Å), and moganite (3.33–3.39 Å), with interplanar spacings matching established crystallographic databases (e.g., AMSCD, ICDD) (Figure 6 and Figure 7). In particular, SAED patterns from these samples clearly exhibited diffraction spots and ring patterns consistent with polycrystalline aggregates of tridymite, cristobalite, and moganite (Figure 8 and Figure 9), in agreement with the Rietveld-identified phase assemblages at this temperature.

The presence of moganite in some nanocrystals, despite its nearly complete disappearance according to bulk XRD quantification, suggests that trace remnants or transitional domains may still exist at the nanoscale—highlighting the added sensitivity of HRTEM in identifying minor or incipient phase domains. The detection of interplanar distances compatible with both monoclinic (I2/a) and orthorhombic (Imab) moganite supports the possibility of a partial symmetry transformation during heating, in line with the thermal behavior described by Heaney and Post [36].

**Figure 10:**
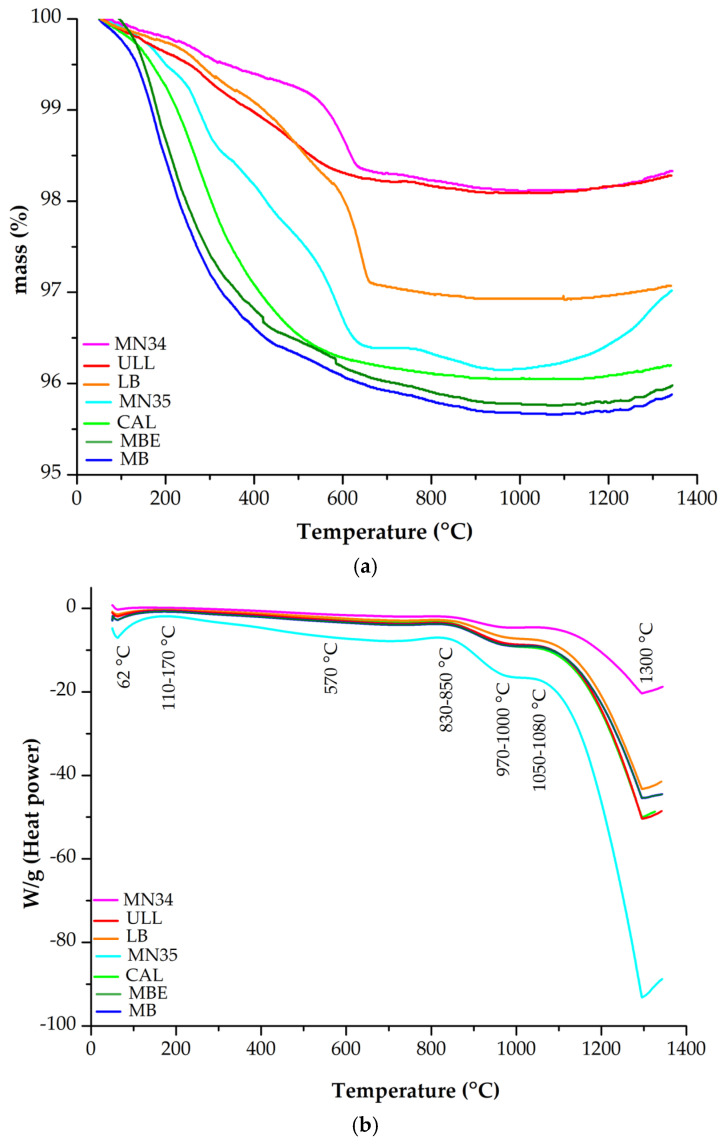
Curves TG (**a**) and DSC (**b**) of the cherts analyzed. In the DSC thermograms, endothermic peaks appear as downward deflections, whereas exothermic peaks are recorded as upward deflections.

The thermal behavior of the investigated samples (MN35, MN34, LB, ULL, MBE, MB, and CAL) is shown in Figure 10. The TG curves (Figure 10a) show that the decomposition of the samples took place in several steps. The first steps extend from 25 to about 250 °C and are due to the loss of adsorbed water on the surface. In the rest of the steps, an additional mass loss is observed besides a slight mass gain towards 1350 °C (Table 4). In the DSC curves of the analyzed cherts, endothermic and exothermic peaks are observed (Figure 10b). The endothermic peaks absorb energy and indicate events such as melting, vaporization, and some chemical reactions. The exothermic peaks indicate events as chemical reactions, oxidation, and crystallization.

The differential thermogravimetric (DTG) profiles of the investigated cherts are provided in the Figure S2 (Supplementary Materials). The curves revealed sample-specific thermal responses associated with mass loss processes across the 50–1400 °C range. MN35 and MB displayed marked DTG peaks between 700 and 900 °C, indicating significant thermally induced transformations. MN34 and ULL exhibited broader peaks around 600–750 °C, whereas CAL showed a delayed response with a gradual weight loss beyond 1000 °C. In contrast, the DTG curve for LB remained relatively flat, reflecting a low reactivity under thermal conditions. These patterns reflect distinct structural and compositional differences among the samples, particularly in relation to the presence of thermally labile phases such as moganite, dolomite, and minor clay components.

Since DSC measurements were performed at a single heating rate, traditional kinetic models such as Kissinger [35] or Ozawa–Flynn–Wall [37,38] could not be applied. Instead, a localized Arrhenius approximation was used, assuming the DSC signal is proportional to the reaction rate. The steps taken to refine the estimation were as follows: (1) Detection of key thermal events (endothermic/exothermic) using polynomial fitting in each temperature interval. (2) Centering the analysis around the peak temperature (±10 °C) to ensure signal relevance. (3) Interpolation and smoothing (cubic spline) of the ln(heat flow) vs. 1/T data to reduce noise. The results are summarized in Table 5; E_a_ could not be estimated in the investigated samples due to insufficient positive data for the fit.

### 3.2. Mechanochemically Treated Cherts

After mechanochemical treatment of the MBE and CG samples, a chromatic analysis was carried out on the mechanochemically treated samples at different grinding durations to assess the visual changes induced by the treatment. As reflected in the chromaticity coordinates Table 6, the CG series exhibited a marked shift from a vivid reddish-orange tone in the untreated sample (CG: x = 0.5015, y = 0.3921) toward progressively more muted and darker hues with increasing treatment time, culminating in CG-24 (x = 0.3718, y = 0.4018), which presented a less-saturated and earthier color. This shift is accompanied by a decrease in luminance (Y), indicating a visual darkening. Conversely, the MBE samples show a subtler evolution in tone, transitioning from a light beige shade in the untreated state (MB: x = 0.3235, y = 0.3506, Y = 0.673) to a slightly darker and less-luminous color after 24 h of treatment (MB-24: x = 0.3238, y = 0.3593, Y = 0.490).

**Figure 11:**
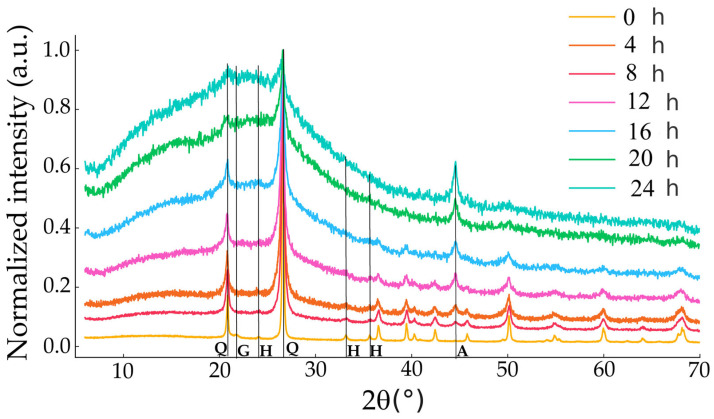
X-ray diffraction patterns of the mechanochemically treated sample CG for 4, 8, 12, 16, 20, and 24 h showing the most characteristic reflections of the identified phases: Q = quartz (100), (101); G = goethite (130); H = hematite (012), (104), (110); A = anorthite ($\overset{-}{4}$13).

**Figure 12:**
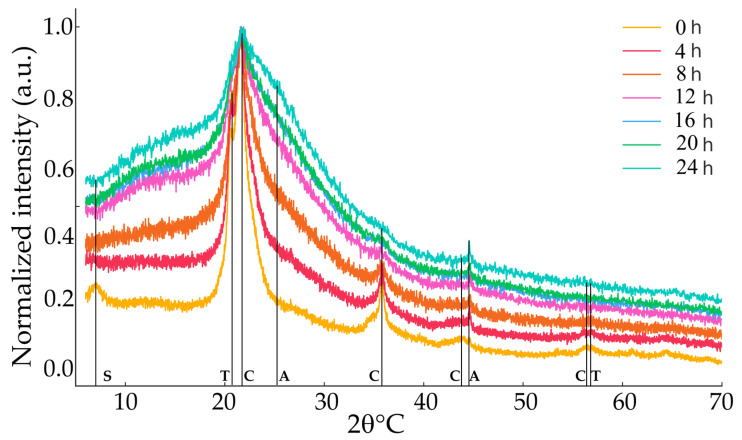
X-ray diffraction patterns of the mechanochemically treated sample MBE for 4, 8, 12, 16, 20, and 24 h showing the most characteristic reflections of the identified phases: S = sepiolite (100); T = trydimite (100), (211); C = cristobalite (111), (220), (222), (331); A = albite (241), ($\overset{-}{1}\bar{1}2$).

X-ray diffraction patterns of the mechanochemically treated samples CG and MBE are presented in Figure 11 and Figure 12, respectively, showing the evolution of the diffracted signal with increasing treatment time. The mineral phases identified in the CG samples and their corresponding ICDD cards were as follows: quartz (ICDD card 46-1045) and h-quartz for the samples CG-20 and CG-24 (ICDD card 11-252), gismondine (ICDD card 39-1373) and microcline (ICDD card 19-926) for the CG-8 sample, anorthite for the rest of the samples, except for the CG, CG-4, and CG-8 samples, and hematite (ICDD card 24-72) and goethite (ICDD card 2-281) for the untreated sample CG. After only 4 h of high-energy milling, a broadening of the peaks and partial amorphization of quartz and accessory silicates was evident. The hematite and goethite peaks disappeared in the first 8 h, which is consistent with the topotactic susceptibility of Fe-oxyhydroxides to impact-induced disordering. Quartz reflections remained detectable until 16 h, while weak reflections of gismondine (110) and anorthite (002) melted into the amorphous halo. Above 16 h the diffractograms were dominated by humps. The mineral phases identified in the MBE samples and their corresponding ICDD cards were cristobalite (ICDD card 27-605), tridymite (ICDD card 16-152), opal (ICDD card 38-448), albite (ICDD card 20-554), orthoclase (ICDD card 8-48), anorthite (ICDD card 10-379), sepiolite (ICDD card 29-1492). The MBE sample showed cristobalite, tridymite, and sepiolite; the sample MBE-4 showed cristobalite, tridymite, and opal; the samples MBE-8 and MBE-12 showed tridymite and opal; the samples MBE-16, MBE-20, and MBE-24 showed albite, orthose, and anorthite. Mechanical activation caused amorphization with the disappearance of cristobalite and the appearance of opal. From 4 to 16 h, the broadening and attenuation of the reflections indicate a reduction of the coherent domain size and an increase of the lattice deformation. Above 16 h, the system reached a mechanically stable amorphous state, with only weak feldspar reflections persisting.

The corresponding crystallinity values, calculated from the diffraction data, are summarized in Table 7. At the beginning of the mechanochemical treatment, the GC sample exhibited higher crystallinity than the MBE. However, after 4 h, MBE showed greater crystallinity than GC. In both samples, crystallinity decreased over time, following a pattern that can be divided into three stages: (1) an initial stage of high crystallinity from 0 to 4 h; (2) a plateau stage from 4 to 8 h, where crystallinity remained lower but relatively stable; and (3) a final stage marked by a pronounced decline at 12 h, leading to significantly lower crystallinity values at 16, 20, and 24 h compared to the initial state.

## 4. Discussion

The compositional homogeneity of the cherts suggests that the thermal transformation processes follow similar pathways in samples with comparable mineralogical features. The influence of mineral composition on thermal response is evidenced by the presence of accessory phases, which may alter the thermal stability of silica [13,14,17,34], as observed in sample MN35.

While some GOF with the Rietveld refinement values appear elevated (e.g., 12 for CAL, MB, MN35 samples or 14 for CAL-1000 samples), this does not reflect analytical errors but rather the intrinsic limitations of the Rietveld model when applied to highly heterogeneous materials such as chert. The microstructural complexity of these samples—characterized by epitaxial intergrowths of quartz and moganite, polymorph coexistence (quartz, cristobalite, tridymite), and abundant structural defects—results in diffraction peak broadening and overlapping that cannot be fully resolved by conventional refinement algorithms. Despite these challenges, the refinements remain reliable for comparative purposes, as all samples were processed under identical experimental conditions. The relative changes in crystallite size and diffraction profile evolution thus provide valuable information on the structural modifications induced by thermal treatment. Structural data obtained through Rietveld refinement of the diffractograms indicate that heating to 1000 °C results in quartz remaining the dominant phase; moganite remains in some samples such as LB and CAL, according to Flörke et al. and Miehe and Graetsch [23,24], and from 900 to 1000 it transforms into cristobalite; while small amounts of high-temperature polymorphs—particularly cristobalite and, to a lesser extent, tridymite—begin to emerge, marking the onset of structural rearrangements within the silica matrix. At 1400 °C, a significant shift in the mineral assemblage occurs: cristobalite becomes the unique phase. These transformations align with the known SiO_2_ phase diagram, in which cristobalite is the stable polymorph at high temperatures, especially in the presence of fluxes or impurities. The thermal stability field of cristobalite is further favored by the fine grain size and high surface area typical of cryptocrystalline silica.

Structural modifications in crystallite size and microstrain parameters indicate recrystallization processes and stress relaxation induced by thermal annealing [39]. These transitions not only provide insight into the thermally induced evolution of siliceous materials but also have implications for the identification of heat-altered lithic artifacts in archaeological contexts and industrial applications such as production of ceramics and sanitary materials [34]. The structural evolution of cristobalite with increasing temperature reveals a progressive recrystallization process. Crystallite size increase at this stage reflects the formation of well-ordered, relaxed crystals.

In summary, thermal treatment promotes structural ordering and recrystallization across all silica phases studied. Quartz undergoes a transient collapse at 1000 °C, followed by recovery at higher temperatures. Tridymite forms as a low-strain, intermediate phase with limited growth. Cristobalite exhibits the most pronounced structural changes, with significant crystallite growth and strain reduction at 1400 °C. These results, consistent with those of Fenner [10] and Mackenzie [11], help reconstruct thermal histories in chert materials. The structural evolution of cristobalite, in particular, may serve as a marker of high-temperature exposure. Differentiation among silica polymorphs is thus key to identifying heat-altered lithic artifacts and understanding associated geological processes. The formation of tridymite and cristobalite can be used to adjust the thermal expansion of ceramic bodies, which helps to avoid defects such as cracks due to mismatches between the ceramic body and the glaze. The formation of the high-temperature silica phases (tridymite and cristobalite) depends on mineralizing agents (NaOH and KOH), together with grain size, temperature, and treatment time, according to Dapiaggia et al. [34]. This allows more efficient and predictable processes to be designed. The presence of agents such as those mentioned above allows the formation of the high temperature phases at significantly lower temperatures than those required for pure quartz, which can reduce energy costs in manufacturing. The proportion of tridymite, cristobalite, and amorphous material depends on the mineralogical composition of the chert, which influences the type of ceramic materials and their specific thermal properties.

The sharp decrease in crystallinity observed for sample LB-1000 °C, from 65.91% to 50.28%, represents a unique behavior among all analyzed specimens. This phenomenon may be attributed to phase transformation processes, particularly the ongoing quartz-to-cristobalite conversion, supported by the presence of high-cristobalite and tridymite phases in the thermally treated samples (Table 2). At this transitional stage, the structure may become partially disordered or even transiently amorphous. The subsequent recovery in crystallinity to 70.66% at 1400 °C suggests that 1300 °C constitutes a critical threshold at which the original structure becomes destabilized. Sample-specific factors, such as the presence of impurities influencing the transformation pathway, may contribute to this behavior. Additionally, thermal stress during heating could generate microcracks or lattice defects, resulting in a temporary reduction in crystallinity. This distinctive transformation pattern highlights the complexity of silica phase transitions and underscores the importance of temperature control during thermal treatment.

From a practical standpoint, the ability to modulate crystallinity through thermal treatment holds promise for ceramic manufacturing, enabling the optimization of specific crystalline phases for applications requiring tailored properties. Phase stability at high temperatures is also critical in refractory materials and glass production [40,41].

The presence of stable quartz–moganite assemblages in the samples MN34, LB, ULL, MB, and CAL—characterized by high initial crystallinity and minimal structural alteration upon heating—is consistent with advanced recrystallization and limited detrital input. In contrast, samples with elevated Al_2_O_3_ or MgO contents (ULL and MBE) and low initial crystallinity undergo marked structural reorganization upon heating, suggesting that structural impurities facilitate silica phase transformations during both diagenesis and thermal activation.

The observed crystallinity trends across the thermally treated samples reflect not only the degree of structural order, but also the onset of amorphization during phase transitions. In particular, abrupt decreases in crystallinity—such as that recorded for sample LB at 1000 °C—may indicate partial structural collapse or the presence of non-diffracting amorphous domains resulting from phase destabilization or microstructural stress. These findings underscore the value of crystallinity as a proxy for tracking structural disorder, even though absolute quantification of amorphous content remains a methodological challenge.

The PCA results further indicate that crystallinity patterns in cherts are not solely determined by silica content but are also influenced by the type and abundance of impurities incorporated during formation. The principal component analysis (Figure 2a) revealed significant differences among the samples. These differences reflect not only variations in initial crystallinity and silica phase composition (e.g., quartz/moganite ratios) but also differences in minor and trace element contents (e.g., Al_2_O_3_, MgO), which—as discussed in the literature [15,16]—can act as structural stabilizers or transformation catalysts for tridymite and cristobalite. Furthermore, Figure 2a illustrated the multivariate relationships between chemical composition and crystallinity behavior across temperatures. Samples such as MBE and MB, with elevated MgO and lower initial crystallinity, contrast sharply with MN35 and MN34, which are richer in SiO_2_ and exhibit sharper quartz peaks and higher starting crystallinity. These compositional and structural contrasts enrich the study by highlighting how naturally embedded variability in chert affects silica phase transformations, which was one of the central goals of this research.

The decision to focus HRTEM investigations on the 1000 °C condition was deliberate. At this intermediate stage, the coexistence of quartz, tridymite, cristobalite, and moganite offers a unique opportunity to resolve and distinguish multiple silica polymorphs in situ. In contrast, the untreated cherts (dominated by quartz) and the fully transformed samples at 1400 °C (exclusively cristobalitic) provide limited structural variability at the nanoscale, which can be more comprehensively characterized by conventional diffraction and thermal methods. Furthermore, the selection of the 1000 °C stage is especially informative because it bridges the gap between the initial mineralogical state and the end product at 1400 °C. It reflects a complex phase environment where both parent and product phases coexist in variable proportions, depending on the nature of each chert. In this context, the application of HRTEM becomes not only relevant but essential to capture the subtleties of ongoing silica phase transformations that are no longer detectable in the simpler end-member states. HRTEM and SAED analyses of the LB-1000 and MN34-1000 samples, heated at 1000 °C, corroborated the formation of tridymite and cristobalite. Tridymite would have formed before cristobalite [10], although according to Dapiaggi [34], tridymite would have formed after cristobalite, and Stevens et al. [13] stated that tridymite would not be a stable phase, and cristobalite would have formed earlier. Furthermore, on the basis of HRTEM observations of sample MN34-1000, it would be possible for monoclinic and orthorhombic phases of moganite to coexist as well, since according to Heaney and Post [36], the transition from the lower (I2/a) to the higher (Imab) symmetry phase would take place around 300 °C. The interplanar distances of 3.33 Å and 3.39 Å could correspond to monoclinic moganite (ICDD 38-360), but the distances 3.36 Å and 3.39 Å could also correspond to orthorhombic moganite (AMCSD 2737). The interplanar distance of 3.33 Å could also correspond to quartz (ICDD 5-490). The interplanar distance of 3.45 Å could correspond to cristobalite (CMSD 20170).

Slight mass gains observed above 1300 °C (~0.15–0.22%) in several samples may be attributed to oxidation of trace impurities (e.g., Fe^2+^ or complex oxides), oxygen incorporation during late-stage structural transitions (e.g., cristobalite crystallization), or instrumental baseline drift at high temperatures. The absence of a corresponding endothermic signal argues against adsorption, indicating a multifactorial origin. Endothermic DSC peaks were observed at several key temperatures: At ~62 °C, the peak likely corresponds to desorption of physically adsorbed water, release of loosely bound structural water, or minor relaxation of metastable phases (e.g., cristobalite or tridymite). The ~570 °C peak is consistent with the α- to β-quartz transition. Peaks near 970–1000 °C may reflect formation of hexagonal tridymite from quartz and cubic cristobalite from moganite. The 1300 °C peak likely corresponds to cubic cristobalite formation, corroborated by XRD patterns of heated cherts. Exothermic DSC peaks were recorded at 110–170 °C, 830–850 °C, and 1050–1080 °C. The 110–170 °C signal may result from dehydration in MB and minor rearrangements in other samples. At 830–850 °C, sepiolite transformed into a glassy phase, and dolomite (in MN35) decomposed, releasing CO_2_. Simultaneously, quartz transformation to tridymite or cristobalite may release heat, as could reactions from trace phases. At 1050–1080 °C, dolomite decomposition continues, and the formation of high-temperature silica polymorphs (tridymite and cristobalite) from quartz or moganite releases additional energy. The estimated activation energy of 0.56 kJ/mol for sample MN35 is associated with weak processes such as desorption of water or minor structural relaxation, rather than major phase transitions. These results are consistent with previous findings by other authors [10,12,13], who documented quartz–tridymite transformations around 1000 °C and subsequent formation of cristobalite near 1300 °C. The transformation of moganite to cubic cristobalite between 900 and 1000 °C also corroborates previous research [23,24].

Several thermal events recorded during DSC analysis showed significant heat flow intensities, notably in samples MN35, MN34, and LB. Events with peak heat flow exceeding ±1.0 W/g suggest pronounced endothermic or exothermic activity, likely related to structural reorganizations or mineral transformations. For instance, the pronounced endothermic peak at 1296 °C in sample LB may reflect high-temperature phase transitions such as quartz inversion or early cristobalite nucleation. The thermal intensity indicates that they could play a key role in the transformation pathways of the silica phases involved. The consideration of peak heat flow is therefore essential not only to identify relevant transitions but also to prioritize events for future multi-rate kinetic modeling.

The DTG profiles confirm and expand upon the phase transformations inferred from DSC and XRD analyses. Samples MB and MN35 exhibit strong DTG peaks in the 700–900 °C range, consistent with exothermic DSC events and XRD evidence for the growth of cristobalite and the transformation of dolomite. MN34 and ULL show broader DTG signals near 650–750 °C, which correlate with the formation of tridymite and the progressive conversion of moganite. CAL presents a delayed response with weight loss occurring above 1000 °C, reflecting a late-stage structural reorganization, possibly due to persistent moganite or low-crystallinity silica. LB, dominated by quartz, shows minimal thermal reactivity, in line with its stability observed in XRD and its weak thermal response in DSC. Collectively, the DTG data support the mineralogical differentiation among the cherts and reinforce the sequence of silica phase evolution identified through structural and thermal techniques.

Mechanochemical activation transformed chert into an increasingly amorphous, highly reactive material with implications significant for both industrial and geological contexts. In industrial applications, including ceramics, glass manufacturing, and construction, the specific silica polymorphs present affect thermal stability, mechanical performance, and reactivity [42]. Chert behaves as a pozzolanic supplementary cementitious component, boosting strength and durability in blended cements; it could be a promising adsorbent or catalytic support for environmental remediation and chemical synthesis. The homogenized composition favors low-temperature sintering and vitrification, enabling its use as a precursor for ceramics and silica-based glass-ceramics; it could be employed as a filler or reinforcing agent in polymer and resin composites. In nature, chert offers insight into the thermal and diagenetic history of cherts [43]. The mechanochemical treatment accelerates natural deformation and pressure-driven processes that promote transformation of unstable silica phases into more stable ones. Mechanical energy in experimental settings induces these reactions, facilitating the transformation of cristobalite into opal, as in the MBE samples. Both tectonic and experimental energy inputs generate defects, fractures, and reactive surfaces that drive the conversion of less-stable phases. The disappearance of cristobalite, together with the formation of opal while retaining tridymite, mimics the typical diagenetic evolution of silica. These mechanisms parallel natural diagenesis, wherein unstable silica dissolves and reprecipitates as more stable forms over geological timescales. For example, cristobalite (typically forming at 1470–1710 °C) can develop at lower temperatures (~1000 °C) in natural systems due to impurities and defects. Its high-temperature stability makes it relevant in refractory materials. From a geoarchaeological standpoint, the treated material could simulate natural weathering of prehistoric chert tools, providing insights into diagenetic pathways and lithic degradation processes.

The mechanochemical treatment produced a striking color change in the GC samples: what was originally a reddish tone became progressively grey as the treatment time progressed. This chromatic change reflects the disappearance of hematite and goethite, responsible for the initial red hue, whose crystalline structures were lost during the high-energy impacts and related amorphization processes.

Mechanochemical treatments shed light on the transformation behavior of silica under stress, revealing the complex interplay among mechanical energy, chemical environment, and mineral stability. These findings enhance our understanding of both natural processes in silica-rich sediments—such as the transformation of opal-CT to quartz in deep-sea cherts—and controlled phase transitions in industrial materials science.

## 5. Conclusions

This study demonstrates that heating cherts up to 1400 °C transforms quartz, moganite, and tridymite into cristobalite, significantly varying the crystallinity of the samples. This result is consistent with the stabilization of cristobalite as the dominant phase at high temperatures and with the completion of polymorphic transitions, which favors the reorganization of the crystal structure. These changes were more pronounced in cristobalitic/tridymitic cherts, which showed substantial increases in crystallinity, while quartzitic cherts exhibited minimal increases or even decreases in crystallinity. These results demonstrate that the proportion of tridymite and cristobalite can be used to adjust the thermal expansion of ceramic materials, which in turn affects both their classification and their specific thermal properties. On the other hand, chert often contains other mineral phases, other than siliceous phases, in a very small proportion, but sufficient for high-temperature phases to form at significantly lower temperatures than those required for pure quartz, which can reduce energy costs in manufacturing of ceramic materials.

Prolonged mechanochemical treatments induced amorphization of phases such as cristobalite and the formation of opal, while quartz remained stable due to its inherent resilience. These transformations are driven by mechanical energy disrupting weaker structures and hydration processes stabilizing the newly formed opal phases. These findings are highly relevant for industries such as ceramics and glass manufacturing, where controlled transformations of silica phases impact the thermal stability, mechanical strength, and reactivity of materials. Additionally, this research provides valuable insights into the thermal history and diagenetic processes of silica-rich rocks, enhancing our understanding of their geological evolution. Mechanochemical treatment provides a rapid simulation of diagenetic evolution, offering insights into the mineralogical transformations that occur over much longer geological timescales. In general, the sensitivity of silica phases to heat and mechanical energy highlights the interconnection of natural processes and industrial applications. From a geoarchaeological standpoint, the mechanochemically treated material could simulate natural weathering of prehistoric chert tools, providing insights into diagenetic pathways and lithic degradation processes.

## Figures and Tables

**Table 1:** Chemical composition (%) of the investigated cherts. The standard deviation (SD) was less than 0.3 for SiO_2_, less than 0.5 for Al_2_O_3_, less than 0.1 for MgO, and less than 0.005 for the other analyzed oxides.

Sample	Oxides (%)										L.O.I (%)
	SiO_2_	CaO	Al_2_O_3_	K_2_O	Fe_2_O_3_	MgO	SO_3_	SrO	Sm_2_O_3_	CuO	
MN35	92.98	1.28	4.75	0.16	0.81	n.d.	0.02	n.d.	n.d	n.d.	3.87
MN34	94.31	1.11	2.46	0.99	0.86	n.d.	0.02	n.d.	0.25	n.d.	1.89
LB	99.97	n.d.	n.d.	n.d.	n.d.	n.d.	0.02	n.d.	n.d.	n.d.	2.40
ULL	89.33	0.17	9.43	0.62	0.43	n.d.	0.02	0.01	n.d.	n.d.	1.92
MB	93.72	0.27	4.81	n.d.	0.06	1.15	n.d.	n.d.	n.d.	n.d.	4.78
MBE	87.87	n.d.	4.05	n.d.	0.07	8.01	n.d.	n.d.	n.d.	0.01	4.50
CAL	99.92	0.07	n.d.	n.d.	n.d.	n.d.	0.01	n.d.	n.d.	n.d.	3.95
CG	80.39	0.08	9.07	0.43	7.48	2.52	0.03	n.d.	n.d.	n.d.	2.35

**Table 2:** Phases identified and their corresponding ICDD card in the investigated samples.

Samples	Phases and (ICDD)		
	Untreated	1000 °C	1400 °C
MN35	Quartz (5–490) Dolomite (36–426)	Quartz (46–1045) Tridymite (16–152)	Cristobalite tetragonal (39–1425)
MN34	Quartz (5–490) Moganite (38–360) Dolomite (36–426)	Quartz (5–490) Quartz (46–1045) Tridymite (3–227)	
LB	Quartz (5–490) Moganite (38–360)	Quartz (46–1045) Moganite (38–360) Cristobalite cubic (27–605) Tridymite (16–152)	
ULL	Quartz (5–490) Moganite (38–360) Muscovite (25–649)	Quartz (46–1045) Cristobalite cubic (27–605) Tridymite (16–152)	
MB	Quartz (5–490) Moganite (38–360) Tridymite (18–1170)	Quartz (46–1045) Cristobalite cubic (27–605) Tridymite (16–152)	
MBE	Cristobalite cubica (27–605) Tridymite (18–1170) Sepiolite (25–1371)	Quartz (46–1045) Cristobalite cubic (27–605) Tridymite (16–152)	
CAL	Quartz (5–490) Moganite (38–360) Cristobalite cubic (27–605)	Quartz (46–1045) Moganite (38–360) Cristobalite cubic (27–605)	

**Table 3:** Crystallinity (%) of untreated and treated samples at 1000 °C and 1400 °C and the differences, Δ (%), between the samples treated at 1000 °C and untreated samples, samples treated at 1400 °C and untreated samples, and samples treated at 1400 °C and samples treated at 1000 °C.

SAMPLE	CRYSTALLINITY (%)			Δ %		
	Untreated	1000 °C	1400 °C	1000 °C	1400 °C	1400–1000 °C
MN35	70.2	72.9	73.4	3.9	4.6	0.5
MN34	69.1	71.7	82.8	3.7	19.8	11.2
LB	65.9	50.3	70.7	−23.7	7.2	20.4
ULL	51.6	69.8	72.5	35.4	40.7	2.7
MB	31.2	32.5	75.8	4.2	143.3	43.3
MBE	26.3	27.4	70.7	4.4	169.1	43.2
CAL	34.1	54.8	59.5	60.5	74.6	4.8

**Table 4:** Loss and gain mass (%) of the cherts with T (°C).

Sample	Loss Mass (%)	T (°C)	Gain Mass (%)	T (°C)
MN35	0.51	201.4	0.86	1350
	0.91	316.9		
	0.80	460.9		
	1.41	706.1		
	0.24	961.5		
MN34	0.18	180	0.22	1350
	0.36	357.7		
	1.17	713.3		
	0.18	1010.6		
LB	0.95	365.1	0.21	1353
	0.87	735.3		
	0.15	985.8		
ULL	0.93	364.87	0.19	1350
	0.86	734.7		
	0.13	985		
MB	0.52	100	0.20	1350
	4.26	1000		
MBE	0.53	100	0.20	1345
	4.10	990		
MB	0.52	100	0.20	1350
	4.26	1000		
CAL	3.95	984	0.15	1350

**Table 5:** Significant thermal events by peak heat flow. Note: The heat flux at the peak corresponds to the heat absorbed or released.

Sample	Event Type	T Peak (°C)	Peak Heat Flow (W/g)
MN35	endothermic	61.91	−6.9175
MN34	endothermic	64.29	−0.2632
	exothermic	129.9	0.1815
	endothermic	995.19	−4.6165
LB	endothermic	1296	−43.2500
	exothermic	662	–2.7210
	endothermic	63	–1.4430
ULL	endothermic	63	−1.9000
	exothermic	169	–0.5249
MB	endothermic	61.93	−2.724
	exothermic	172.12	−0.7553
MBE	endothermic	54.44	−466.4605
	exothermic	172.12	−0.7553
CAL	endothermic	62.93	−1.2541
	exothermic	168.42	−0.2868

**Table 6:** Chromatic coordinates for the mechanochemically treated samples.

Samples	x	y	Y_lum	Color
CG	0.501536	0.392090	0.184579	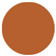
CG-4	0.386332	0.392474	0.256278	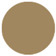
CG-8	0.406310	0.404646	0.250196	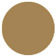
CG-12	0.380335	0.400864	0.188412	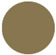
CG-16	0.367416	0.394015	0.301335	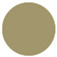
CG-20	0.359847	0.396264	0.289927	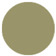
CG-24	0.371816	0.401806	0.269814	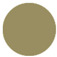
MBE	0.323468	0.350641	0.672891	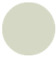
MBE-24	0.323792	0.359266	0.489721	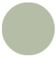

**Table 7:** Crystallinity values (%) of the mechanochemically treated CG and MBE samples at different treatment times, calculated from the X-ray diffraction patterns.

Samples	Time (h)	Crystallinity (%)
CG	0	75.31
CG-1	1	42.84
CG-4	4	19.83
CG-8	8	19.04
CG-12	12	6.26
CG-16	16	4.26
CG-20	20	3.98
CG-24	24	3.38
MBE	0	60.97
MBE-1	1	32.34
MBE-4	4	34.98
MBE-8	8	24.27
MBE-12	12	13.15
MBE-16	16	9.98
MBE-20	20	9.82
MBE-24	24	9.64

## Data Availability

The original contributions presented in this study are included in the article/supplementary material. Further inquiries can be directed to the corresponding authors.

## Supplementary Materials

The following supporting information can be downloaded at: https://www.mdpi.com/article/10.3390/ma18133077/s1, Figure S1: X-ray diffraction patterns of the untreated cherts and cherts heated at 1000 °C and 1400 °C of the samples MN34, LB, ULL, and MB showing the principal reflections of the identified phases; Figure S2: Curves DTG of the cherts analyzed; Table S1: Rietveld data.
