# Supplementary material for: Phase Stability and Structural Reorganization of Silica in Cherts Under Thermal and Mechanochemical Stress
Source: Materials (Basel). 2025 Jun 28;18(13):3077. doi: 10.3390/ma18133077 (PMC12250848; doi:10.3390/ma18133077)
Supplement: Supplementary file 1 [file materials-18-03077-s001.zip › materials-3694180-supplementary figures.docx]

| 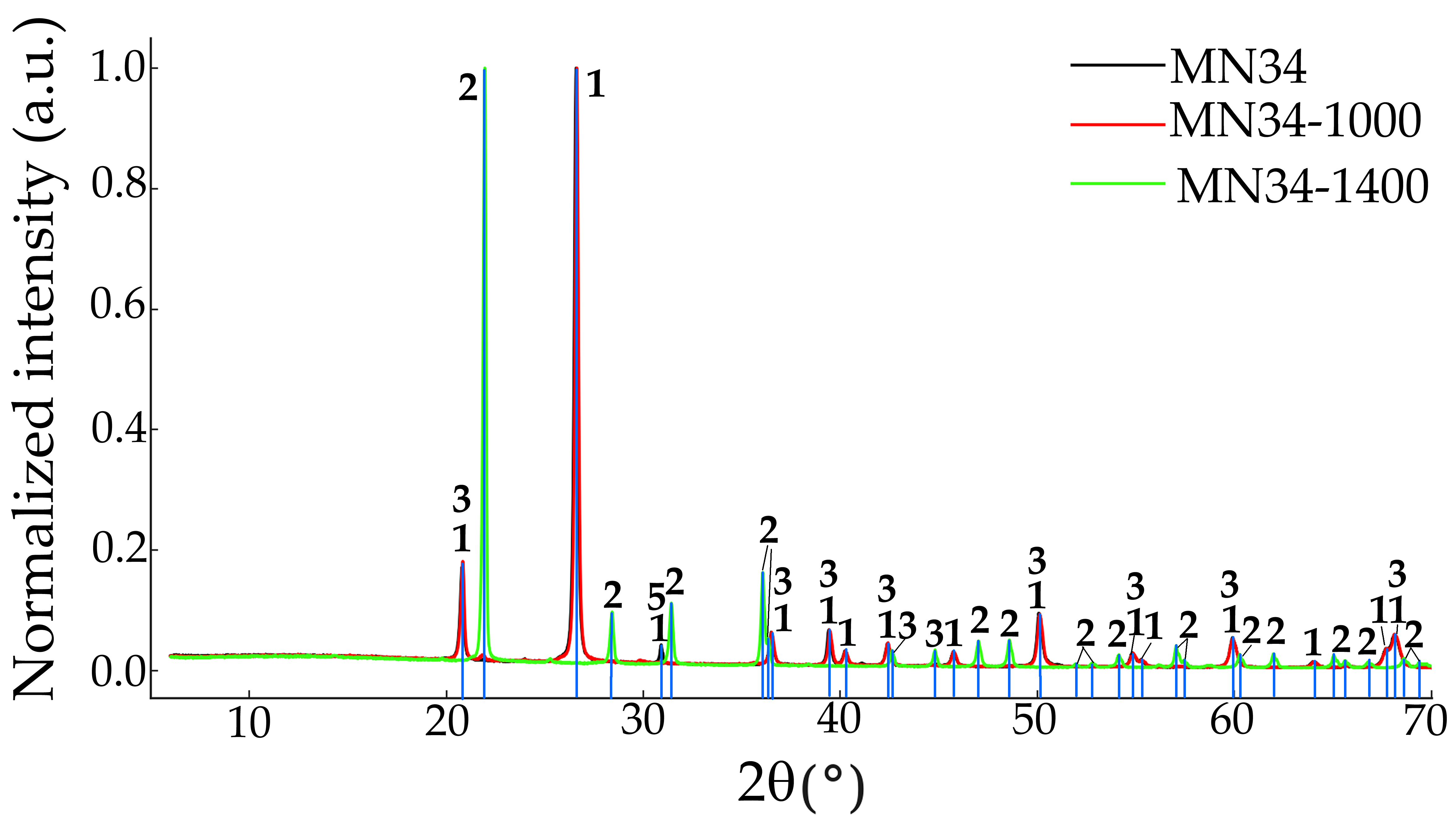 |
| --- |
| 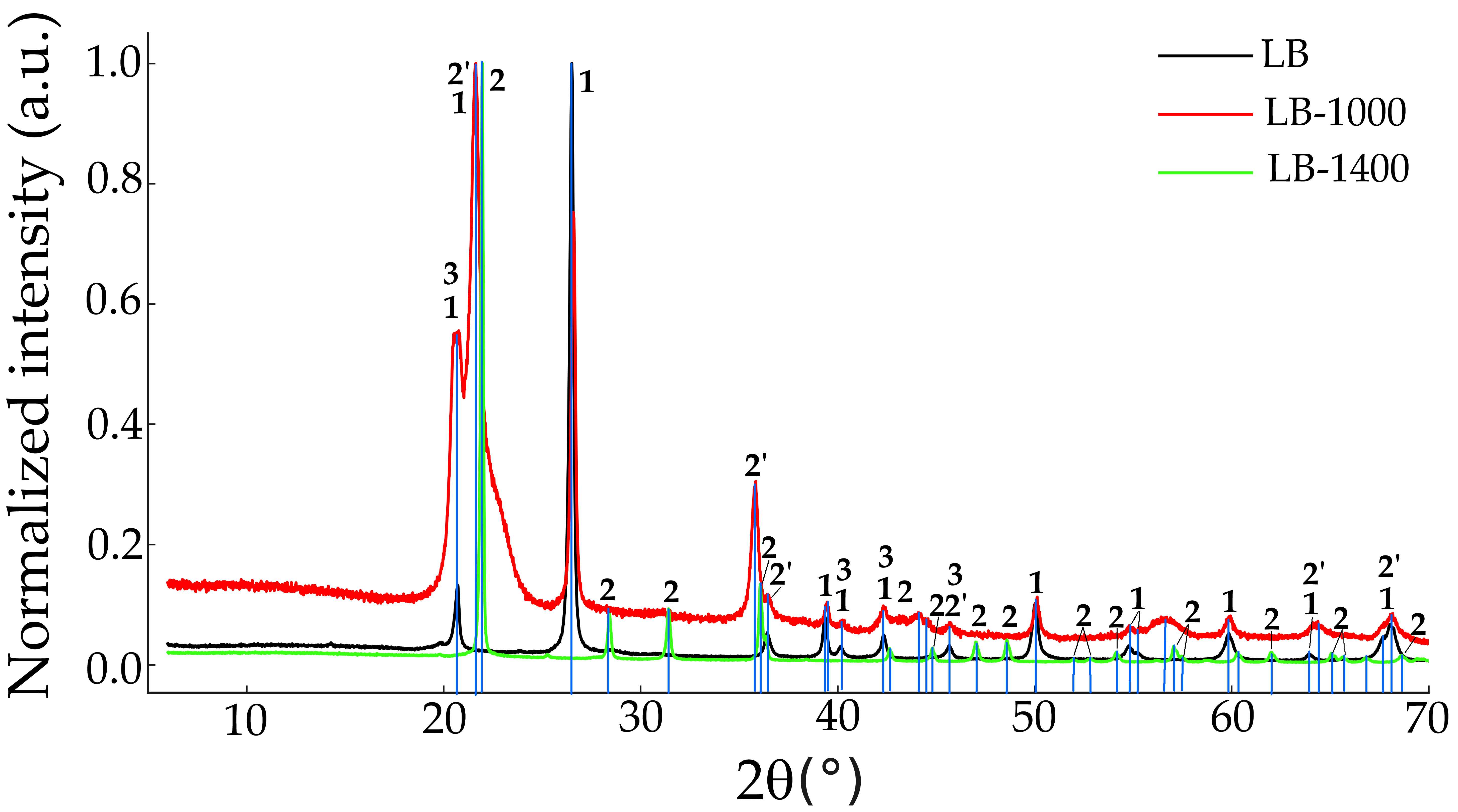 |
| 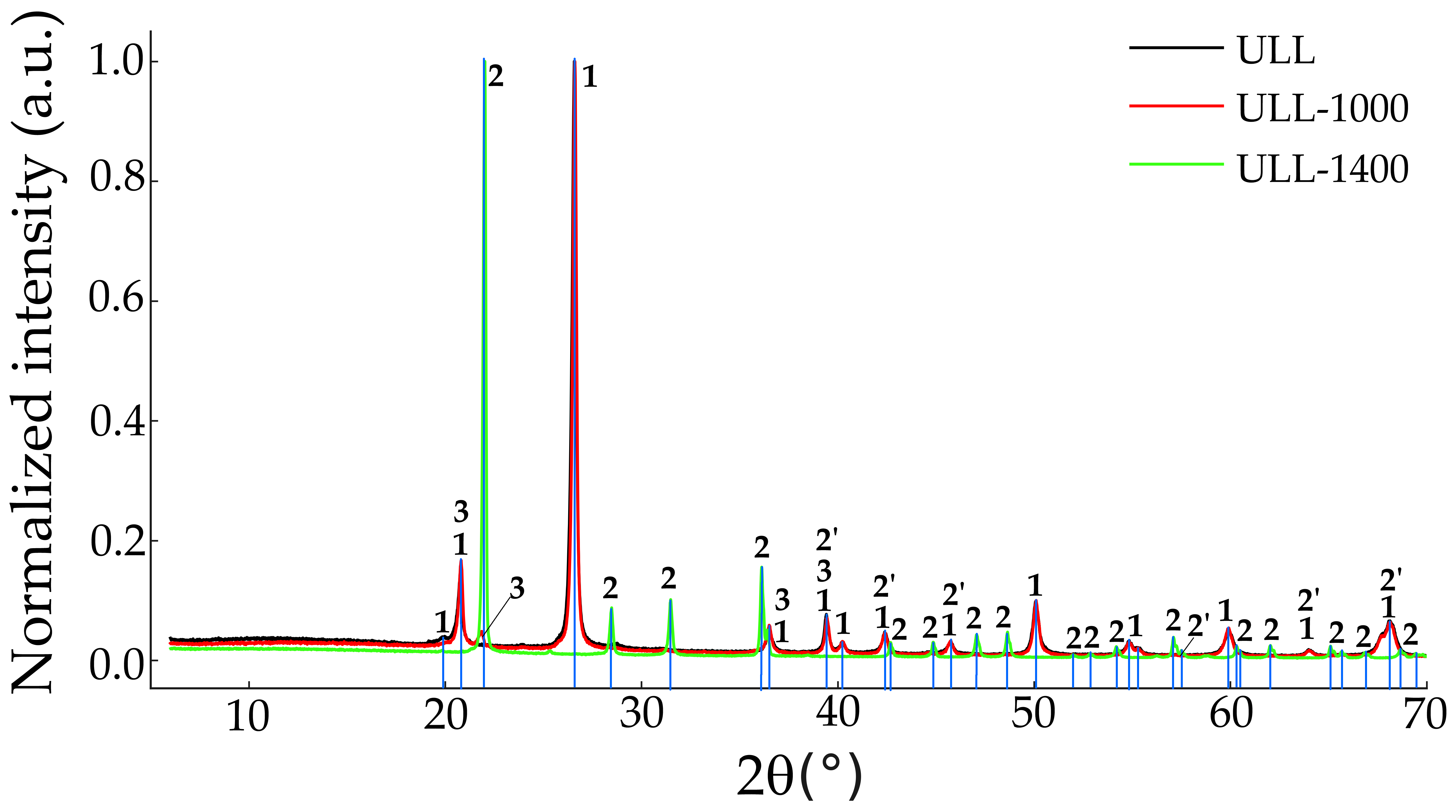 |
| 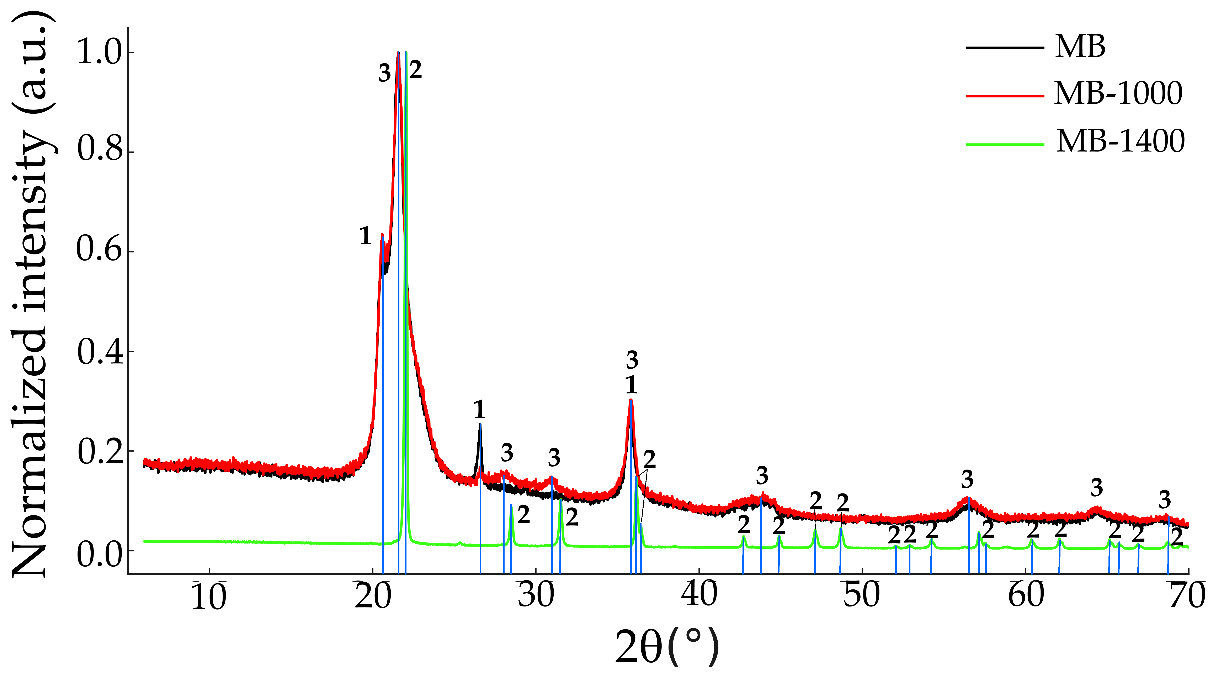 |

**Figure S1.** X-ray diffraction patterns of the untreated cherts and cherts heated at 1000 °C and 1400 °C of the samples MN34, LB, ULL, and MB showing the principal reflections of the identified phases. In the sample NM34: 1 = quartz (100), (101), (110), (102), (111), (201), (112), (103), (211), (203); 2 = cristobalite tetragonal (101), (111), (102), (112), (211), (202), (113), (212), (203), (311), (302), (312), (214), (105); 3 = tridymite overlapping with quartz reflections; 5 = dolomite (104). In the sample LB: 1 = quartz with the same reflections as NM34; moganite overlapping with quartz; 2’ = cristobalite cubic (111), (220) plus reflections overlapping with those of quartz; 2= cristobalite tetragonal with the same reflections as NM34; 3 = tridymite overlapping with quartz reflections. In the sample ULL: 1 = quartz with the same reflections as NM35; moganite overlapping with quartz and perhaps some muscovite, with reflections overlapping those of quartz; 2’ = cristobalite cubic (111), (220) plus reflections overlapping with those of quartz; 2 = cristobalite tetragonal with the same reflections as NM34; 3 = trydimite (115), (203), (220), (2,0,15), (2,2,10). In the sample MB: 1 = quartz (100), (101), (113), (203) overlapping with moganite and coinciding with 3 = trydimite (112), ($\bar{4}03$), (020), ($\bar{7}14$), ($\bar{6}$,0,10), ($\bar{8}08$), (136); 2 = cristobalite cubic (111), (220).

|  |
| --- |
| 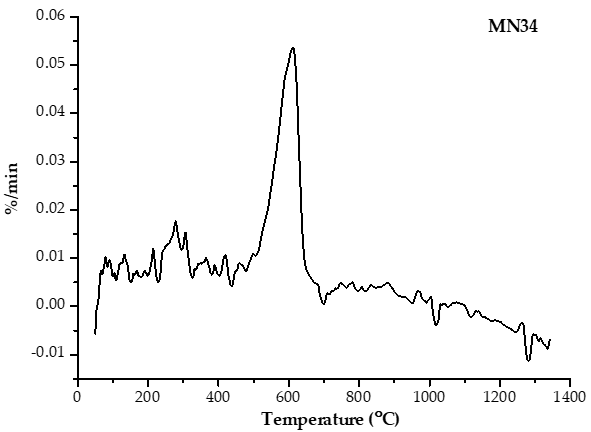 |
| 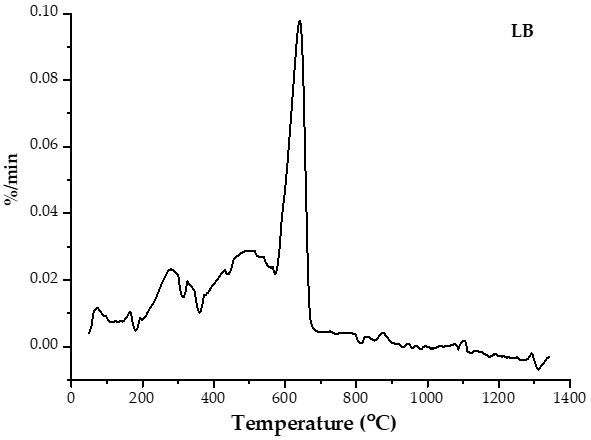 |
| 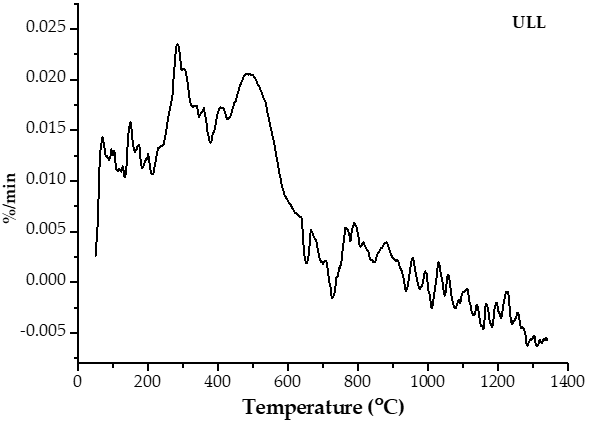 |
| 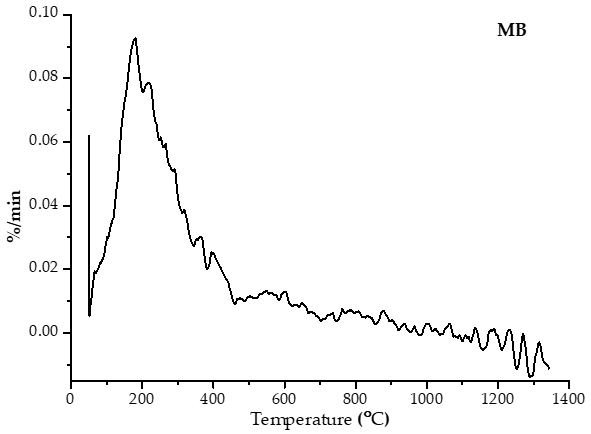 |
| 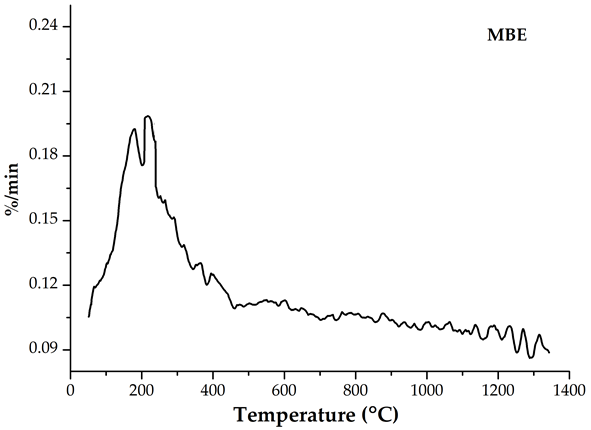 |
| 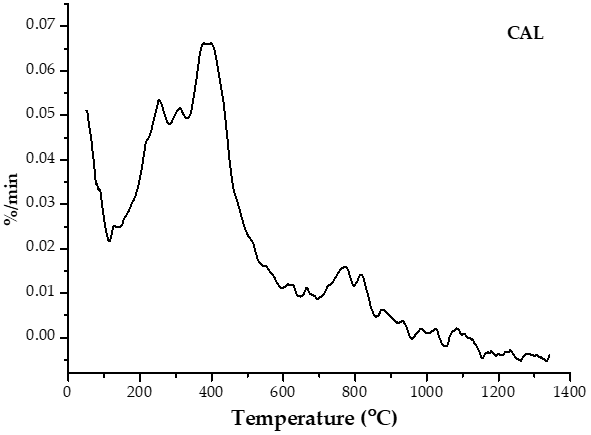 |

**Figure S2**. Curves DTG of the cherts analyzed.
