# Supplementary material for: Phase Stability and Structural Reorganization of Silica in Cherts Under Thermal and Mechanochemical Stress
Source: Materials (Basel). 2025 Jun 28;18(13):3077. doi: 10.3390/ma18133077 (PMC12250848; doi:10.3390/ma18133077)
Supplement: Supplementary file 1 [file materials-18-03077-s001.zip › materials-3694180-supplementary Table S1.docx]

**Table S1**: Rietveld data

| LB | | | | | | | | | | | | |
| --- | --- | --- | --- | --- | --- | --- | --- | --- | --- | --- | --- | --- |
|  | Quartz | | | Moganite | | | Tridymite | | | Cristobalite | | |
|  | Un-treated | 1000 ºC | 1400 ºC | Un-treated | 1000 ºC | 1400 ºC | Un-treated | 1000 ºC | 1400 ºC | Un-treated | 1000 ºC | 1400 ºC |
| a (Å) | 4.910336  (557) | 4.910929  (1274) |  | 8.532023  (5931) | 8.543014  (5931) |  |  | 18.50995  (7057) |  |  | 5.009499  (1482) | 4.969321  (117) |
| b (Å) | 4.910336  (557) | 4.910929  (1274) |  | 4.90225  (3242) | 4.93116  (3242) |  |  | 5.001685  (2162) |  |  | 5.009499  (1482) | 4.969321  (117) |
| c (Å) | 5.399063  (593) | 5.411399  (2106) |  | 10.69431  (6736) | 10.78868  (6736) |  |  | 24.52934  (9376) |  |  | 7.111526  (6338) | 6.926045  (239) |
| α (°) | 90 | 90 |  | 90 | 90 |  |  | 90 |  |  | 90 | 90 |
| β (°) | 90 | 90 |  | 90.14238(192231) | 89.84298(101649) |  |  | 107.7161(26159) |  |  | 90 | 90 |
| γ (°) | 120 | 120 |  | 90 | 90 |  |  | 90 |  |  | 90 | 90 |
| V (Å) | 112.7383 | 113.02 |  | 453.22 | 454.59 |  |  | 2163.252 |  |  | 178.4643 | 171.0328 |
| Crystallite size (Å) | 378 | 369.5 |  | >1000 | 129.7 |  |  | 218.6 |  |  | 181.4 | 1055 |
| Micro Strain (%) | 0.126 | - |  | - | - |  |  | - |  |  | - | 0.112 |
| Content (%) | 99.5 | 20 |  | 0.5 | 13.4 |  |  | 29.2 |  |  | 37.4 | 100 |
| ULL | | | | | | | | | | | | |
|  | Quartz | | | Moganite | | | Tridymite | | | Cristobalite | | |
|  | Un-treated | 1000 ºC | 1400 ºC | Un-treated | 1000 ºC | 1400 ºC | Un-treated | 1000 ºC | 1400 ºC | Un-treated | 1000 ºC | 1400 ºC |
| a (Å) | 4.911823(231) | 4.918174(342) |  | 9.445715(6044) |  |  |  | 5.065463(18967) |  |  | 7.036958(2572) | 4.971083(95) |
| b (Å) | 4.911823(231) | 4.918174(342) |  | 3.874605(1999) |  |  |  | 5.065463(18967) |  |  | 7.036958(2572) | 4.971083(95) |
| c (Å) | 5.399655(317) | 5.400447(45) |  | 16.67074(28545) |  |  |  | 13.12983(45826) |  |  | 7.036958(2572) | 6.924661(232) |
| α (°) | 90 | 90 |  | 90 |  |  |  | 90 |  |  | 90 | 90 |
| β (°) | 90 | 90 |  | 102.1996(4062) |  |  |  | 90 |  |  | 90 | 90 |
| γ (°) | 120 | 120 |  | 90 |  |  |  | 120 |  |  | 90 | 90 |
| V (Å) | 112.819 | 113.1275 |  | 596.3444 |  |  |  | 291.7616 |  |  | 348.4616 | 171.1199 |
| Crystallite size (Å) | 441 | 580.2 |  | > 1000 |  |  |  | 27.9 |  |  | 361.4 | 816 |
| Micro Strain (%) | 0.146 | 0.177 |  | 0 |  |  |  | 0 |  |  | 0.028 | 0.039 |
| Content (%) | 98.4 | 75.9 |  | 1.6 |  |  |  | 21.9 |  |  | 2.2 | 100 |
| CAL | | | | | | | | | | | | |
|  | Quartz | | | Moganite | | | Tridymite | | | Cristobalite | | |
|  | untreated | 1000 ºC | 1400 ºC | untreated | 1000 ºC | 1400 ºC | untreated | 1000 ºC | 1400 ºC | untreated | 1000 ºC | 1400 ºC |
| a (Å) | 5.011082(2233) | 4.916561 |  | 8.437064(16332) |  |  |  | 17.73639 |  | 5.011716(1978) |  | 4.969273(11) |
| b (Å) | 5.011082(2233) | 4.916561 |  | 4.99273(13409) |  |  |  | 5.074614 |  | 5.011716(1978) |  | 4.969273(11) |
| c (Å) | 5.320318(2075) | 5.40172 |  | 13.63119(27948) |  |  |  | 25.70162 |  | 7.272538(10018) |  | 6.930155(222) |
| α (°) | 90 | 90 |  | 90 |  |  |  | 90 |  | 90 |  | 90 |
| β (°) | 90 | 90 |  | 78.74013(173118) |  |  |  | 106.3664 |  | 90 |  | 90 |
| γ (°) | 120(201) | 120 |  | 90 |  |  |  | 90 |  | 90 |  | 90 |
| V (Å) | 115.6994 | 113.0799 |  | 563.1474 |  |  |  | 2219.546 |  | 182.6665 |  | 171.131 |
| Crystallite size (Å) | 226 | 774.1 |  | > 1000 |  |  |  | 103 |  | 355 |  | 1021 |
| Micro Strain (%) | 0 | 0.221 |  | 0 |  |  |  | - |  | 1.168 |  | 0.143 |
| Content (%) | 50.9 | 89.2 |  | 12 |  |  |  | 10.8 |  | 37 |  | 100 |
| MB | | | | | | | | | | | | |
|  | Quartz | | | Moganite | | | Tridymite | | | Cristobalite | | |
|  | untreated | 1000 ºC | 1400 ºC |  |  |  | untreated | 1000 ºC | 1400 ºC | untreated | 1000 ºC | 1400 ºC |
| a (Å) | 4.589873(5094) | 4.744701(7249) |  |  |  |  | 17.8171(12211) | 18.503760(14892) |  | 5.065879(2993) | 5.031922(2227) | 4.970339(91) |
| b (Å) | 4.589873(5094) | 4.744701(7249) |  |  |  |  | 5.087918(2808) | 5.022676(4909) |  | 5.065879(2993) | 5.031922(2227) | 4.970339(91) |
| c (Å) | 6.191195(1294) | 5.660258(22324) |  |  |  |  | 24.80926(18012) | 24.74500(18944) |  | 7.514956(5591) | 7.162752(10478) | 6.923759(185) |
| α (°) | 90 | 90 |  |  |  |  | 90 | 90 |  | 90 | 90 | 90 |
| β (°) | 90 | 90 |  |  |  |  | 106.7335(61225) | 107.86440(51674) |  | 90 | 90 | 90 |
| γ (°) | 120 | 120 |  |  |  |  | 90 | 90 |  | 90 | 90 | 90 |
| V (Å) | 112.9553 | 110.351 |  |  |  |  | 2153.772 | 2189.09 |  | 192.8573 | 181.35 | 171.0464 |
| Crystallite size (Å) | 116.8 | 55.6 |  |  |  |  | 95.6 | 140.8 |  | 132.3 | 107.1 | 856 |
| Micro Strain (%) | - | 0.851 |  |  |  |  | - | 0.101 |  | - | 0.238 | 0.052 |
| Content (%) | 2.5 | 20.6 |  |  |  |  | 49.6 | 18.4 |  | 47.9 | 60.9 | 100 |
| MBE | | | | | | | | | | | | |
|  | Quartz | | | Moganite | | | Tridymite | | | Cristobalite | | |
|  | untreated | 1000 ºC | 1400 ºC |  |  |  | untreated | 1000 ºC | 1400 ºC | untreated | 1000 ºC | 1400 ºC |
| a (Å) |  | 4.744701(7249) |  |  |  |  | 17.949660 (32354) | 18.749550(41263) |  | 5.028511(717) | 5.001723(3336) | 4.970354(108) |
| b (Å) |  | 4.744701(7249) |  |  |  |  | 5.285478(6841) | 5.203753(5321) |  | 5.028511(717) | 5.001723(3336) | 4.970354(108) |
| c (Å) |  | 5.660258(22324) |  |  |  |  | 23.755620(50283) | 22.643682(31276) |  | 7.412665(8936) | 7.231842(20458) | 6.92148(225) |
| α (°) |  | 90 |  |  |  |  | 90 | 90 |  | 90 | 90 | 90 |
| β (°) |  | 90 |  |  |  |  | 114.229200(140523) | 112.976154(232432) |  | 90 | 90 | 90 |
| γ (°) |  | 120 |  |  |  |  | 90 | 90 |  | 90 | 90 | 90 |
| V (Å) |  | 110.351 |  |  |  |  | 187.4361 | 186.183 |  | 185.4361 | 171.35 | 170.9911 |
| Crystallite size (Å) |  | 55.6 |  |  |  |  | 46.3 | 69.8 |  | 46.3 | 30.1 | 753 |
| Micro Strain (%) |  | 0.851 |  |  |  |  | 0.635 | 0.364 |  | 0.635 | 0.738 | 0.041 |
| Content (%) |  | 10.2 |  |  |  |  | 39.70 | 30.41 |  | 60.20 | 60.39 | 100 |
| MN34 | | | | | | | | | | | | |
|  | Quartz | | | Moganite | | | Tridymite | | | Cristobalite | | |
|  | Un-treated | 1000 ºC | 1400 ºC | Un-treated | 1000 ºC | 1400 ºC | Un-treated | 1000 ºC | 1400 ºC | Un-treated | 1000 ºC | 1400 ºC |
| a (Å) | 4.912537(112) | 4.917488(288) |  |  |  |  |  | 5.047910(1967) |  |  |  | 4.971564(102) |
| b (Å) | 4.912537(112) | 4.917488(288) |  |  |  |  |  | 5.047910(1967) |  |  |  | 4.971564(102) |
| c (Å) | 5.402237(89 | 5.401055(444) |  |  |  |  |  | 8.117571(5521) |  |  |  | 6.92485(213) |
| α (°) | 90 | 90 |  |  |  |  |  | 90 |  |  |  | 90 |
| β (°) | 90 | 90 |  |  |  |  |  | 90 |  |  |  | 90 |
| γ (°) | 120 | 120 |  |  |  |  |  | 120 |  |  |  | 90 |
| V (Å) | 112.9057 | 113.1086 |  |  |  |  |  | 179.135 |  |  |  | 171.1577 |
| Crystallite size (Å) | 636.6 | 723.1 |  |  |  |  |  | > 1000 |  |  |  | 666 |
| Micro Strain (%) | 0.145 | 0.173 |  |  |  |  |  | - |  |  |  | 0.05 |
| Content (%) | 99.4 | 99.6 |  |  |  |  |  | 0.4 |  |  |  | 100 |
| MN35 | | | | | | | | | | | | |
|  | Quartz | | | Moganite | | | Tridymite | | | Cristobalite | | |
|  | Un-treated | 1000 ºC | 1400 ºC |  |  |  | Un-treated | 1000 ºC | 1400 ºC | Un-treated | 1000 ºC | 1400 ºC |
| a (Å) | 4.912120(138) | 4.914775(102) |  |  |  |  |  | 8.73705 |  |  |  | 4.970539(92) |
| b (Å) | 4.912120(138) | 4.914775(102) |  |  |  |  |  | 11.133 |  |  |  | 4.970539(92) |
| c (Å) | 5.402611(191) | 5.401885(114) |  |  |  |  |  | 6.912586 |  |  |  | 6.923829(192) |
| α (°) | 90 | 90 |  |  |  |  |  | 90 |  |  |  | 90 |
| β (°) | 90 | 90 |  |  |  |  |  | 90 |  |  |  | 90 |
| γ (°) | 120 | 120 |  |  |  |  |  | 90 |  |  |  | 90 |
| V (Å) | 112.8944 | 113.0013 |  |  |  |  |  | 672.3845 |  |  |  | 171.069 |
| Crystallite size (Å) | 662.0 | 730.3 |  |  |  |  |  | 27.9 |  |  |  | 1029 |
| Micro Strain (%) | 0.034 | 0.065 |  |  |  |  |  | 0 |  |  |  | 0.062 |
| Content (%) | 98.4 | 88.5 |  |  |  |  |  | 11.5 |  |  |  | 100 |
